# The effect of primary health care on tuberculosis in a nationwide cohort of 7·3 million Brazilian people: a quasi-experimental study

**DOI:** 10.1016/S2214-109X(21)00550-7

**Published:** 2022-01-24

**Authors:** Gabriela S Jesus, Julia M Pescarini, Andrea F Silva, Ana Torrens, Wellington M Carvalho, Elzo P P Junior, Maria Y Ichihara, Mauricio L Barreto, Poliana Rebouças, James Macinko, Mauro Sanchez, Davide Rasella

**Affiliations:** a Faculty of Medicine, Federal University of Bahia, Salvador, Brazil; b Institute of Collective Health, Federal University of Bahia, Salvador, Brazil; c Centre for Data and Knowledge Integration for Health, Instituto Gonçalo Moniz, Fundação Oswaldo Cruz, Salvador, Brazil; d Vital Strategies, Civil Registration and Vital Statistics Improvement and Data Impact Programs, São Paulo, Brazil; e Pan-American Health Organization, WHO Country Office for Brazil, Brasilia, Brazil; f Departments of Health Policy and Management and Community Health Sciences, University of California, Los Angeles Fielding School of Public Health, Los Angeles, CA, USA; g Department of Public Health, University of Brasilia, Brasilia, Brazil; h ISGlobal, Hospital Clínic— Universitat de Barcelona, Barcelona, Spain

## Abstract

**Background:**

Universal health coverage is one of the WHO End TB Strategy priority interventions and could be achieved—particularly in low-income and middle-income countries—through the expansion of primary health care. We evaluated the effects of one of the largest primary health-care programmes in the world, the Brazilian Family Health Strategy (FHS), on tuberculosis morbidity and mortality using a nationwide cohort of 7·3 million individuals over a 10-year study period.

**Methods:**

We analysed individuals who entered the 100 Million Brazilians Cohort during the period Jan 1, 2004, to Dec 31, 2013, and compared residents in municipalities with no FHS coverage with residents in municipalities with full FHS coverage. We used a cohort design with multivariable Poisson regressions, adjusted for all relevant demographic and socioeconomic variables and weighted with inverse probability of treatment weighting, to estimate the effect of FHS on tuberculosis incidence, mortality, cure, and case fatality. We also performed a range of stratifications and sensitivity analyses.

**Findings:**

FHS exposure was associated with lower tuberculosis incidence (rate ratio [RR] 0·78, 95% CI 0·72–0·84) and mortality (0·72, 0·55–0·94), and was positively associated with tuberculosis cure rates (1·04, 1·00–1·08). FHS was also associated with a decrease in tuberculosis case-fatality rates, although this was not statistically significant (RR 0·84, 95% CI 0·55–1·30). FHS associations were stronger among the poorest individuals for all the tuberculosis indicators.

**Interpretation:**

Community-based primary health care could strongly reduce tuberculosis morbidity and mortality and decrease the unequal distribution of the tuberculosis burden in the most vulnerable populations. During the current marked rise in global poverty due to the COVID-19 pandemic, investments in primary health care could help protect against the expected increases in tuberculosis incidence worldwide and contribute to the attainment of the End TB Strategy goals.

**Funding:**

TB Modelling and Analysis Consortium (Bill & Melinda Gates Foundation), Wellcome Trust, and Brazilian Ministry of Health.

**Translation:**

For the Portuguese translation of the abstract see Supplementary Materials section.

## Introduction

Tuberculosis is among the ten leading causes of death worldwide and is considered a poverty-related disease; a high tuberculosis burden is frequently observed in populations with low socioeconomic status, with restricted access to food and precarious housing conditions.^1^ Tuberculosis morbidity and mortality in low-income and middle-income countries (LMICs) are also concentrated in men and people of working age (>14 years). During the COVID-19 pandemic, a substantial increase in the worldwide tuberculosis burden has been predicted due to the disruption of tuberculosis health services and an increase in poverty, especially in LMICs.^2^ Therefore, rapid and widespread interventions are urgently needed to mitigate the effects of the pandemic on tuberculosis.

According to the WHO End TB Strategy, the expansion of universal health coverage (UHC) is one of the most feasible and effective interventions that could reduce the tuberculosis burden by 2025.^3^ As reaffirmed in the recent Declaration of Astana,^4^ primary health care should be considered the cornerstone of universal health coverage and its expansion and improvement could represent the fastest way to get closer to this goal.

Over the past two decades, Brazil has implemented one of the world's largest and most effective primary health care interventions, the Family Health Strategy (FHS), which is one of the most widely evaluated primary health care programmes. The FHS is based on family health teams, comprising one doctor, nurses, and community health workers who are responsible for health promotion, prevention, health-care assistance, recovery, and follow-up of patients and individuals in their catchment areas (around 3450 inhabitants), including through home visits and community interventions (appendix 2 p 3).^5^ Studies have shown that FHS can reduce infant and child mortality, hospitalisations from conditions sensitive to ambulatory care, and heart and cerebrovascular disease mortality.^5^ Nevertheless, after a large expansion over the past decade and attainment of 64% coverage of the Brazilian population in 2019,^6^ FHS expansion has stagnated because of fiscal austerity measures.^7^

**Research in context**

**Evidence before this study**

Primary health care, as a cornerstone of universal health coverage, is considered a priority intervention to reduce the world's tuberculosis burden according to the WHO End TB Strategy. To investigate the available evidence, we searched PubMed and Embase for studies published in any language between Jan 1, 2000, and May 25, 2021, containing the following terms: “primary health care” OR [MeSH Terms] “primary care” [MeSH Terms] AND “tuberculosis” [MeSH Terms]. We also verified referenced studies from the selected articles. We found 1863 studies, and among them only four evaluated the association between primary health care and tuberculosis morbidity and mortality. All studies were done in Brazil and all but one had an ecological design, preventing interpretations in terms of causal inference. The only individual-level observational study that evaluated the effect of the Brazilian Family Health Strategy on several mortality causes, among them tuberculosis, was based only in one Brazilian city and found a 55% reduction in tuberculosis mortality among individuals exposed to primary health care. The other ecological-level studies showed a negative association between primary health care coverage and tuberculosis incidence and mortality, and a positive association with tuberculosis cure rates, with imperfect interpretability due to—among other reasons—ecological fallacy.

**Added value of this study**

To our knowledge, as of the date of this study, no other studies have used a cohort with such a large number of individuals over an extended period of time, combined with a robust quasi-experimental design, to evaluate in such a comprehensive way the effect of one of the world's largest primary health-care strategies on sequential indicators of tuberculosis morbidity and mortality. Using a nationwide cohort with 7 308 968 individuals followed up for 10 years, 7184 tuberculosis cases, a causal inference framework of analysis, multivariable regressions adjusted for the relevant demographic and socioeconomic characteristics, inverse probability treatment weighting based on the same adjusting variables, and various sensitivity analyses, we found a strong impact of primary health care on reducing tuberculosis incidence, mortality, and case-fatality rates, and increasing tuberculosis cure rates. Moreover, we found that the poorest individuals benefitted most from primary health care, showing the role of primary health care in the reduction of health inequalities. Our results also show the potential of using linked health and socioeconomic administrative datasets to study the effect of nationwide policies on poverty-related diseases.

**Implications of all the available evidence**

Our results, together with other findings and theoretical considerations, present evidence that primary health care is a crucial factor for the attainment of the WHO End TB Strategy goals and is especially important in the context of the current global economic recession due to COVID-19, which is increasing poverty rates and consequently the tuberculosis burden worldwide.

Brazil has one of the highest tuberculosis burdens worldwide, and although there have been considerable improvements in tuberculosis control in Brazil during recent years, the attainment of the Sustainable Development Goal for tuberculosis is unlikely given current trends.^8^ The detection and management of tuberculosis cases in Brazil is decentralised and under the responsibility of the primary level of health care, mainly FHS units. Although a small number of studies—all at the ecological level—showed negative associations between FHS coverage and tuberculosis incidence and mortality,^9^, ^10^ to our knowledge, no large-scale individual-level evaluations have been done to date. We aimed to comprehensively evaluate the effect of the FHS on sequential tuberculosis outcomes, namely incidence, cure rate, mortality, and case-fatality rate, using a large cohort of 7·3 million Brazilian people during the period Jan 1, 2004, to Dec 31, 2013.

## Methods

### Study design

This study had a cohort quasi-experimental design based on longitudinal information from 7·3 million individuals for the years 2004–13. From the 114 million individuals of the 100 Million Brazilians Cohort, we selected a subcohort for this study based on the time period for which all data on tuberculosis were available (Jan 1, 2004, to Dec 31, 2013), and on the degree of coverage by FHS, including only individuals residing in municipalities with no or full FHS coverage during the study period.

This study was done according to the Declaration of Helsinki and Brazilian research regulations and was approved by the research ethics committee of the Federal University of Bahia (NP: 3.910.696). The 100 Million Brazilians Cohort has waived the need for informed consent, as the cohort has been built through the linkage of administrative databases.

### Data sources, linkage, and intervention

The 100 Million Brazilians Cohort, built by the Center for Data Integration and Knowledge in Health (CIDACS),^11^ was based on linkage between health datasets and the Brazilian national record for social assistance—the Cadastro Único (CadUnico)—which represents approximately the poorest 50% of the Brazilian population.^12^, ^13^

Regarding health information, the datasets used were the National Disease Notification System (SINAN) and the Mortality Information System (SIM).^6^ SINAN is a decentralised surveillance system that monitors the incidence of notifiable diseases. SIM is a national mortality information system that registers all deaths and their causes, codified according to International Classification of Disease 10th revision (ICD-10) classification.

Algorithms and codes were developed to make efficient, highly sensitive, and specific linkages using the name of each individual, date of birth, sex, and municipality of residence present in each of these data systems.^11^, ^14^, ^15^ The 100 Million Brazilians Cohort baseline and SINAN datasets were linked by five individual-level identifiers in two steps using the CIDACS-record linkage tool. In the first step, entries were deterministically linked. In the second step, entries that were not linked deterministically were then linked based on a similarity score between all the pairwise comparisons (ie, ranging from 0 to 1); entries with the highest similarity scores were considered to be linked pairs. The quality of each linkage between CadUnico, SINAN, and SIM (for all causes) have been widely evaluated and validated.^16^, ^17^

We evaluated all relevant indicators on the pathway from infection to mortality from tuberculosis (defined by ICD-10 codes A15–19) as follows: tuberculosis incidence (number of new tuberculosis cases divided by person-years × 100 000), tuberculosis cure rate (number of tuberculosis cases with treatment success minus  proven cure plus complete treatment divided by the number of tuberculosis cases × 100), tuberculosis case-fatality (number of deaths from tuberculosis divided by the number of tuberculosis cases × 100), and tuberculosis mortality rates (number of deaths from tuberculosis divided by person-years × 100 000), for the general population and for strata defined by demographic and socioeconomic characteristics, such as income, sex, and age. Further stratifications for education are shown in appendix 2 (p 10).

Annual FHS coverage was calculated according to the Brazilian Ministry of Health definition: the total number of teams deployed in the municipality in that year multiplied by 3450—representing the average number of individuals served by each FHS team—divided by the population of the municipality.^6^ CadUnico records were linked to municipal FHS coverage data by municipality code with deterministic (exact) linkage. The exposed group was defined as individuals residing in municipalities with 100% FHS coverage during the entire study period (2004–13), and the unexposed group consisted of the individuals residing in municipalities with less than 10% FHS coverage during the same period. The threshold of 10% coverage was used to reduce a large imbalance in the ratio of exposed individuals to unexposed individuals, which could have resulted in less reliable effect estimates. As sensitivity analyses, all the models were also evaluated with unexposed individuals defined as only individuals residing in municipalities with no FHS coverage.

### Statistical analysis

To estimate the association between FHS exposure and tuberculosis incidence, mortality, cure and case-fatality rate, we used multivariable Poisson regression models adjusted for all relevant demographic and socioeconomic confounding variables, with follow-up time as an offset variable, and with observations weighted using stabilised truncated inverse probability of treatment weighting (IPTW). The follow-up time was truncated by the end of the study period (Dec 31, 2013), or by the occurrence of the outcome under study, or by the death of the individual.

IPTW Poisson models are widely used in quasi-experimental studies that investigate the effects of public and social policies on health outcomes expressed as rates, and have been used in similar quasi-experimental studies using the CadUnico dataset and the 100 Million Brazilians Cohort.^18^, ^19^, ^20^, ^21^, ^22^ To calculate weights, we first estimated the likelihood that each individual would be covered by FHS with multivariable logistic regression models.^23^, ^24^, ^25^ The independent variables or covariates used in these regressions comprised baseline demographic and socioeconomic characteristics of the individuals who registered in the cohort between 2004 and 2013, were recorded at their baseline status before any tuberculosis diagnosis, and include sex, age, education, race, number of family members, household construction material, per capita family income, period of receipt of the money allowances from the Brazilian conditional cash transfer, (if the family is among its beneficiaries), cumulative municipal tuberculosis incidence over the study period among the individuals of the cohort, and year of entry into the cohort. For cure and case-fatality rates, we also included, as covariates, the comorbidities AIDS and diabetes.

The four tuberculosis outcomes under study had different denominators; although tuberculosis incidence and mortality rates used the entire cohort of 7·3 million individuals as their denominator, the case-fatality rates used only 5368 tuberculosis cases in the cohort with no missing values for any of the independent variables. For tuberculosis cure rate only, the study period was censored at Dec 31, 2011, and the denominator was 3369 tuberculosis cases because of the under-reporting of cured cases between Jan 1, 2012, and Dec 31, 2013 (because of delayed cure). As a second step, we calculated the stabilised inverse probability of treatment weight for individuals not exposed using the formula (1 – P_t_) / (1 – PS_mul_) and for the individuals exposed using the formula P_t_ / PS_mul_, where P_t_ is the marginal probability of treatment in the population and PS_mul_ is the propensity score obtained from the multivariable logistic regression adjusted for all variables. As a third step, to solve the problems that can arise with very extreme weights, we used truncated or trimmed weights, in which the observation weights that exceeded a specified threshold were redefined by this threshold.^23^, ^24^, ^25^ In this study, the specified thresholds were based on the distribution of weights for the first and 99th percentiles. As a fourth and final step, after stabilisation and weight truncation, we fitted the IPTW multivariable Poisson regression with the same comprehensive set of demographic and socioeconomic covariates used in the previous logistic regression. To evaluate the heterogeneity of FHS effect, we repeated the same process and re-estimated specific propensity scores, weights, and weighted Poisson models for different population stratifications, as follows: by income (above and below the median of the distribution), by age (>15 years and ≤15 years), and by sex. We also stratified the analyses on tuberculosis cure rates according to the introduction of the four-drug fixed-dose combination regimen (a new treatment regimen that comprised rifampicin, isoniazid, pyrazinamide, and ethambutol) in 2009 (appendix 2 p 11).

We did several sensitivity analyses (appendix 2 p 9). First, to test if the threshold used for the unexposed individuals affected the estimates, we evaluated the effect of FHS using the same methods but with individuals resident in municipalities with 0% FHS coverage over the entire study period as unexposed. Second, to evaluate the robustness of the results, we used a different quasi-experimental approach—propensity score matching—to estimate FHS effects, and compared these results with those obtained from the IPTW method. Third, to verify the relevance of IPTW use for obtaining unbiased FHS estimates, we ran the same multivariable Poisson regressions without IPTW and compared the results. Fourth, to test the influence of municipal-level endemic levels of tuberculosis, we ran the same regressions without the cumulative municipal tuberculosis incidence as the adjusting variable. Fifth, to verify the importance of adjusting for all covariates in the IPTW Poisson regression, we ran the models as Poisson bivariate IPTW regression between the tuberculosis outcome and FHS exposure. Sixth, to test the relevance of multilevel modelling, we fitted the same IPTW Poisson regressions for tuberculosis incidence and mortality adding relevant municipal-level adjusting variables.

All analyses were done using STATA version 15.0.

### Role of the funding source

The funders of the study had no role in study design, data collection, data analysis, data interpretation, or writing of the report.

## Results

**Figure 1:**
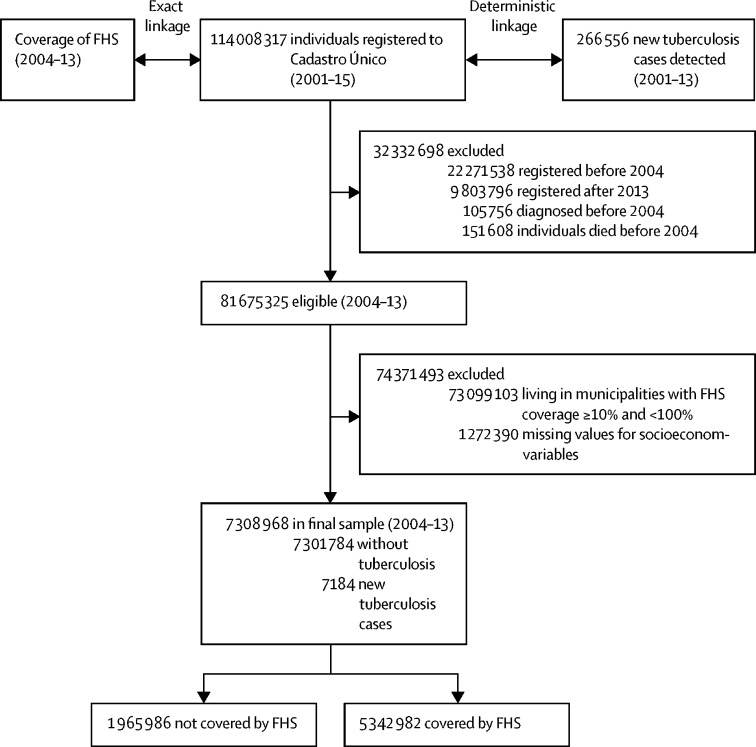
Flowchart of the study cohort (Brazil, 2001–15) FHS=Family Health Strategy.

The baseline cohort taken from the 100 Million Brazilians Cohort contained socioeconomic information from 114 008 317 individuals. In linking this database with SINAN, between Jan 1, 2004, and Dec 31, 2013, 266 556 individuals had tuberculosis (figure 1). Individuals were excluded from the study cohort for the following reasons: being registered in the CadUnico or diagnosed with tuberculosis outside the study period, not living in municipalities with the selected ranges of FHS coverage for the entire study period, and having missing data for a covariate. The final cohort consisted of 7 308 968 individuals— 1 965 986 lived in municipalities with zero (or very low) FHS coverage over the entire study period and 5 342 982 lived in municipalities with full FHS coverage (figure 1). 3 435 215 (47·0%) of 7 308 968 individuals were younger than 15 years and 3 850 625 (53·7%) of 7 308 968 individuals were men.

During follow-up, 7184 new cases of tuberculosis were detected, of which 3007 occurred among individuals living in municipalities with no FHS coverage and 4177 occurred among individuals living in municipalities with full FHS coverage. Among individuals who lived in municipalities with no FHS coverage, the mean incidence rate of tuberculosis in the study period was higher (25·8 per 100 000 person-years) than among individuals who lived in cities with full FHS coverage (12·0 per 100 000 person-years; table 1). The same result was observed in relation to tuberculosis mortality, which was higher among unexposed individuals (0·6 per 100 000 person-years) than among the group with full FHS coverage (0·4 per 100 000 person-years).

**Table 1:** Family Health Strategy coverage

	**Not covered (n=1 965 986)**	**Covered (n=5 342 982)**
Incidence rate per 100 000 person-years*	25·8	12·0
Mortality rate per 100 000 person-years†	0·6	0·4
Cure rate‡	83·3%	84·7%
Case-fatality rate§	2·5%	4·0%

* (Number of new cases/number of individuals) × 100 000.

† (Number of deaths/number of individuals) × 100 000.

‡ (Number of cured/new cases) × 100.

§ (Number of deaths/new cases) × 100.

Residents of exposed municipalities had a higher tuberculosis cure rate (84·7%) than did residents in municipalities with no FHS coverage (83·3%), and the case-fatality rate was higher in municipalities with full coverage (4·0%) than among unexposed individuals (2·5%).

Although most of the demographic and socioeconomic variables were relatively similar between the two groups, there were also meaningful differences in some variables (table 2). Unexposed municipalities had more people of White ethnicity or race and a higher proportion of individuals with higher levels of education than did exposed municipalities. In unexposed municipalities, families were larger and had lower family income per capita (a higher proportion of individuals with an income below the median).

**Table 2:** Individuals covered and not covered by the Family Health Strategy in the 100 Million Brazilians cohort, Brazil, 2004–13.

		**Not covered (n=1 965 986)**	**Coveredn=5 342 982)**
Sex			
	Men	953 721 (48·5%)	2 896 904 (54·2%)
	Woman	1 012 265 (51·5%)	2 446 078 (45·8%)
Age, years		20·5 (18·8)	20·8 (19.3)
Education			
	Never attended	606 170 (30·8%)	1 934 949 (36·2%)
	Primary school or less (<5 years of education)	606 246 (30·8%)	1 807 448 (33·8%)
	Junior high school (≥5 years but ≤9 years of education)	509 060 (25·9%)	1 139 673 (21·3%)
	High school (≥10 years of education)	244 510 (12·4%)	460 912 (8·6%)
Ethnicity or race			
	White	1 115 204 (56·7%)	1 650 996 (30·9%)
	Black	116 378 (5·9%)	338 040 (6·3%)
	Brown	734 404 (37·4%)	3 353 946 (62·8%)
Number of individuals per household			
	≤2	334 487 (17·0%)	1 010 601 (18·9%)
	>2 to ≤4	939 381 (47·8%)	2 527 908 (47·3%)
	≥5	692 118 (35·2%)	1 804 473 (33·8%)
Household material			
	Brick or cement	1 672 352 (85·1%)	3 855 868 (72·2%)
	Other (wood or other vegetal materials)	293 634 (14·9%)	1 487 114 (27·8%)
Family per capita income			
	Below the median (0·5 $BR/month)	1 357 396 (69·0%)	2 573 797 (48·2%)
	Above the median (0·5 $BR/month)	608 590 (31·0%)	2 769 185 (51·8%)
Time receiving Bolsa Família, months		54·48 (41·39)	70·17 (44·38)
Year of entry of individual in cohort			
	2004	155 449 (7·9%)	322 483 (6·0%)
	2005	149 319 (7·6%)	381 286 (7·1%)
	2006	519 499 (26·4%)	2 103 466 (39·4%)
	2007	215 877 (11·0%)	712 847 (13·3%)
	2008	121 191 (6·2%)	316 153 (5·9%)
	2009	135 482 (6·9%)	330 887 (6·2%)
	2010	159 090 (8·1%)	247 657 (4·6%)
	2011	159 167 (8·1%)	270 015 (5·1%)
	2012	203 268 (10·3%)	389 234 (7·3%)
	2013	147 644 (7·5%)	268 954 (5·0%)

Data are n (%) or mean (SD).

The cumulative tuberculosis incidence and mortality from 2004 to 2013 according to FHS coverage are shown in the appendix 2 (pp 27–28). Unadjusted cumulative tuberculosis incidence and mortality among the most socioeconomically vulnerable Brazilian people were markedly lower among individuals residing in municipalities with full FHS coverage than among individuals living in cities with no coverage for the entire study period. Multivariable logistic regression models for the prediction of the propensity scores, including the odds ratios for each predictor, are presented in the appendix 2 (p 8).

Rate ratios (RR) estimated by the IPTW multivariable Poisson regressions, for the total study population and according to stratifications, are shown in Table 3, Table 4. Full FHS coverage was associated with a reduction in tuberculosis incidence (RR 0·78, 95% CI 0·72–0·84; table 3). The strongest FHS effects were observed among the most economically vulnerable individuals, in men, and in younger individuals (table 4).

**Table 3:** Inverse probability of treatment weighting Poisson regression models, adjusted for all demographic and socioeconomic variables, for the association between tuberculosis outcomes and FHS coverage in the study cohort (Brazil, 2004–13)

	**Incidence**	**Mortality**	**Cure rate**	**Case-fatality rate**
FHS 100%	0·78 (0·72–0·84)	0·72 (0·55–0·94)	1·04 (1·00–1·08)	0·84 (0·55–1·30)
Female sex	0·59 (0·55–0·63)	0·40 (0·31–0·51)	1·03 (1·00–1·08)	0·60 (0·35–1·01)
Age, categorised in 10-year age groups	1·41 (1·39–1·43)	1·59 (1·51–1·67)	0·99 (0·97–1·00)	1·19 (1·04–1·37)
<5 years education	1·30 (1·19–1·41)	0·91 (0·72–1·16)	1·01 (0·95–1·07)	0·66 (0·37–1·18)
5 years to ≤9 years education	1·80 (1·64–1·96)	0·76 (0·55–1·06)	1·03 (0·97–1·09)	0·46 (0·20–1·04)
≥10 years education	1·85 (1·64–2·10)	0·46 (0·29–0·75)	1·08 (1·00–1·15)	0·32 (0·11–0·92)
Black ethnicity	1·83 (1·64–2·04)	2·74 (1·94–3·89)	0·95 (0·89–1·02)	1·46 (0·86–2·48)
Brown ethnicity	1·29 (1·20–1·39)	2·09 (1·62–2·69)	1·01 (0·98–1·05)	1·54 (1·01–2·36)
>2 to ≤4 individuals per household	0·82 (0·75–0·90)	0·73 (0·56–0·96)	1·06 (1·00–1·12)	0·50 (0·29–0·84)
≥5 individuals per household	0·96 (0·87–1·06)	0·93 (0·68–1·28)	1·05 (0·99–1·11)	0·88 (0·52–1·49)
Other house material (wood or other vegetal materials)	1·08 (1·01–1·16)	1·20 (0·95–1·53)	0·99 (0·95–1·04)	1·18 (0·75–1·86)
Income above the median	0·88 (0·83–0·95)	0·83 (0·65–1·06)	0·99 (0·95–1·02)	0·84 (0·55–1·29)
In Bolsa Família Program (months)	1·00 (1·00–1·00)	0·99 (0·99–1·00)	1·00 (0·99–1·00)	0·99 (0·99–1·00)
Municipal incidence	1·00 (1·00–1·01)	1·00 (1·00–1·00)	1·00 (0·99–1·00)	0·99 (0·99–1·00)
AIDS	··	··	1·57 (1·35–1·84)	7·82 (1·88–32·40)
Diabetes	··	··	0·94 (0·87–1·02)	0·98 (0·53–1·81)
Observations	7 308 968	7 308 968	3379	5·368

Data are RR (95% CI), unless otherwise indicated. Reference variables were male sex, never attended education, White ethnicity or race, person by domicile ≤2, brick household material, family per capita income below the median (0·5 $BR per month). FHS=Family Health Strategy. RR=rate ratio.

**Table 4:** Inverse probability of treatment weighting Poisson regression models, adjusted for all demographic and socioeconomic variables, for the association between tuberculosis outcomes and the Family Health Strategy coverage according to stratum of income, sex, and age (Brazil, 2004–13)

	**Incidence**	**Mortality**	**Cure rate**	**Case-fatality rate**
**Income**				
Below the median	0·72 (0·65–0·81)	0·71 (0·47–1·06)	1·06 (1·00–1·12)	0·82 (0·44–1·51)
Above the median	0·85 (0·77–0·94)	0·76 (0·54–1·06)	1·02 (0·97–1·07)	0·97 (0·53–1·74)
**Sex**				
Male	0·77 (0·70–0·84)	0·66 (0·49–0·90)	1·05 (0·99–1·10)	0·81 (0·49–1·31)
Female	0·78 (0·69–0·89)	0·87 (0·50–1·49)	1·03 (0·97–1·08)	0·93 (0·39–2·20)
**Age**				
<15 years	0·64 (0·51–0·80)	0·30 (0·11–0·78)	1·01 (0·92–1·11)	0·15 (0·01–1·33)
≥15 years	0·71 (0·66–0·77)	0·73 (0·55–0·96)	1·04 (1·00–1·08)	0·92 (0·60–1·42)

Data are RR (95% CI). All models have been adjusted for the same demographic and socioeconomic variables as in table 3.

FHS coverage was associated with lower mortality rates (RR 0·72, 95% CI 0·55–0·94; table 3). In stratified analyses, the results were similar to those of tuberculosis incidence—the largest FHS effects on mortality were seen among people with income below the median, men, and those younger than 15 years (table 4).

FHS coverage was also associated with higher tuberculosis cure rates (RR 1·04, 95% CI 1·00–1·08; table 3), and a similar effect on cure rates was observed in the different subpopulations, which was stronger among the poorest individuals and men. For tuberculosis case-fatality rates, the RR was 0·84 (95% CI 0·55–1·30), with a stronger association in individuals below the median of income.

Demographic and socioeconomic adjusting variables showed an overall protective effect of being a woman, having a higher level of education, and having a higher income per capita, and an increased risk among Black people, older people, and those living in households built with precarious materials (table 3). The associations for all adjusting variables for the models in table 4 are shown in the appendix 2 (pp 15–18).

## Discussion

In this study, we showed the comprehensive effect of one of the world's largest primary health-care strategies on sequentially measurable tuberculosis outcomes—incidence, mortality, cure, and case-fatality rates. Using a nationwide cohort of 7 308 968 individuals with 7184 tuberculosis cases and a robust quasi-experimental design, we found an association between FHS and reduced tuberculosis incidence and mortality and increased tuberculosis cure rates. FHS was also associated with a decrease in tuberculosis case-fatality rates, although this was not significant. The effect of FHS was stronger among the poorest individuals, highlighting the importance of primary health care in the reduction of health inequalities.^26^

The multidisciplinary teams of FHS—including community health workers that follow up local families for prevention, health promotion, and health education—offer basic health care, referrals, management of risk factors, and home visits.^27^ Several studies have shown that FHS can reduce infant and child mortality, increase access to health care in remote areas, decrease hospitalisations and mortality from conditions sensitive to ambulatory care, and reduce mortality from heath and cardiovascular diseases.^5^

**Figure 2:**
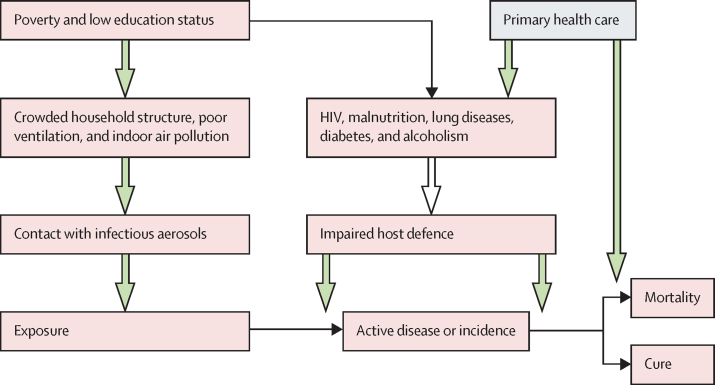
Conceptual framework for the demographic and socioeconomic factors affecting incidence, mortality, cure rate, and fatality rate for tuberculosis, and of the effects of primary health care

FHS activities could increase the detection of new tuberculosis cases in the community and, together with early diagnosis and medical follow-up, could interrupt the tuberculosis chain of transmission in the population (figure 2). FHS has been responsible for the detection of most of the tuberculosis cases and subsequent medical follow up in Brazil during the past two decades, with increasing proportions over the period.^28^

Our findings on the impact of FHS on tuberculosis incidence support those of a population-based ecological study that showed that primary health coverage was associated with a lower incidence of tuberculosis.^10^ In our study, we found a reduction in the tuberculosis mortality rate among individuals covered by the FHS. Although this finding could be the direct consequence of the impact of the FHS on tuberculosis incidence, the larger FHS effect size on mortality than on incidence, and the negative association of FHS with case-fatality rates—although not statistically significant—suggest that the effect of FHS on mortality could be the combined result of its effects on both incidence and case-fatality rate. These findings are compatible with an individual-level cohort analysis that used a similar study design but was focused on the city of Rio de Janeiro, and found a similar impact of FHS coverage on tuberculosis mortality rates,^20^ and with another longitudinal ecological study in all Brazilian municipalities.^9^

Our analyses showed a beneficial effect of the FHS on tuberculosis cure rates, the plausibility of which relies on the ability of the FHS to reduce barriers to health-care access^27^ and consequently improve tuberculosis treatment outcomes. In analyses stratified by income, we found that the most economically vulnerable individuals benefited more from FHS coverage, with greater impacts on tuberculosis incidence, mortality, and cure rate. Previous studies have shown a stronger effect of FHS on the reduction of child mortality in municipalities with a lower human development index,^29^ and other individual-level analyses found larger FHS effectiveness in the reduction of amenable mortality among Black individuals with high baseline mortality rates.^30^ These findings show the potential of primary health care, and in particular FHS, to reduce health inequalities, particularly for conditions and diseases associated with poverty, such as tuberculosis.

Although there has been a decreasing trend in tuberculosis incidence over the past few decades in Brazil, since the economic recession of 2015 tuberculosis incidence has continuously increased,^28^ with a similar trend for extreme poverty over the same period. Although, to our knowledge, the association between increasing tuberculosis morbidity and poverty during this period has not been evaluated, it is plausible that the worsening of socioeconomic conditions contributed to such tuberculosis trends. According to our results, the attainment of full FHS coverage could have mitigated this increase in tuberculosis incidence.

Our study was possible because of the use of nationwide linked datasets and the relatively high endemicity of tuberculosis in Brazil, enabling us to use a cohort of more than 7 million individuals and more than 7000 tuberculosis cases over a 10-year period. Using the large 100 Million Brazilians Cohort, we were able to perform an individual-level impact evaluation that was unprecedented in terms of comprehensiveness and statistical power. Similar research using the 100 Million Brazilians Cohort, and comparable study designs and analytic strategies, has been done for other infectious diseases, such as leprosy. However, to our knowledge, this is the first time that four sequential indicators of a disease—two related to morbidity and two to mortality—were evaluated to understand the effects on their causal pathway.^18^, ^19^, ^20^, ^21^

Our study has limitations. First, although the 100 Million Brazilians Cohort is a powerful source of sociodemographic information, it contains information on the poorest half of the population, so the FHS effect estimates are not necessarily representative of all Brazilian people. Despite this limitation, the cohort includes individuals from all regions of the country. Second, although we were able to estimate the effect of FHS on tuberculosis by adjusting the models for many socioeconomic variables, propensity-score-based models do not control for unobserved confounding. Therefore, additional confounding could still be present. For this reason, and to control for the endemic level of tuberculosis in the municipality, we included the cumulative municipal tuberculosis incidence over the study period as a variable in the adjustment of both logistic and Poisson regression models. Results from sensitivity analyses showed that the inclusion of other variables related to inequalities, infrastructure, and health-care assistance at the municipal level do not affect the FHS effect estimates (appendix 2 p 16). Another limitation is the use of municipalities with FHS coverage less than 10%—instead of 0%—as unexposed over the study period. Although this could have partly distorted the results for unexposed individuals and affected the impact estimates, our sensitivity analyses showed no relevant changes in the effect sizes when municipalities with 0% were used as unexposed (appendix 2 p 12). Another limitation was the different demographic structure of the cohort compared with the general Brazilian population, with 47% of children in the cohort being younger than 15 years (compared with 21% nationally); the over-representation of this age group, which usually has a lower tuberculosis burden, could have affected the reported overall measures of morbidity and mortality of the cohort. However, our stratification analyses showed consistent FHS effects on both age groups.

In conclusion, our findings show that community-based primary health care could reduce morbidity and mortality from tuberculosis and decrease the inequity of tuberculosis burden in an LMIC such as Brazil. In the context of the current global economic recession due to COVID-19, primary health care could mitigate the expected increase in tuberculosis incidence related to the marked rise in poverty rates, representing an important resilience factor during this crisis.

## Data sharing

The protocol for the creation of the 100 Million Brazilians Cohort and the cohort profile of the 100 Million Brazilians Cohort is available in the publications referenced in the article and further material is available at: https://cidacs.bahia.fiocruz.br/en/platform/cohort-of-100-million-brazilians. The linkage protocols are explained in the referenced publications and the codes are available at: https://gitHub.com/gcgbarbosa/cidacs-rl. Individual-level data will not be available for sharing because of confidentiality and ethical issues.

## Declaration of interests

We declare no competing interests.

## Supplementary Materials

Portuguese translation of the abstract

## References

[1] Lönnroth K, Jaramillo E, Williams BG, Dye C, Raviglione M (2009). Drivers of tuberculosis epidemics: the role of risk factors and social determinants. Soc Sci Med.

[2] McQuaid CF, Vassall A, Cohen T, Fiekert K, White RG (2021). The impact of COVID-19 on TB: a review of the data. Int J Tuberc Lung Dis.

[3] WHO (May 16, 2021). The End TB Strategy. https://www.who.int/teams/global-tuberculosis-programme/the-end-tb-strategy.

[4] Kluge H, Kelley E, Barkley S (2018). How primary health care can make universal health coverage a reality, ensure healthy lives, and promote wellbeing for all. Lancet.

[5] Bastos ML, Menzies D, Hone T, Dehghani K, Trajman A (2017). The impact of the Brazilian family health strategy on selected primary care sensitive conditions: a systematic review. PLoS One.

[6] DATASUS Informações em Saúde (TABNET). http://tabnet.datasus.gov.br/cgi/menu_tabnet_php.htm.

[7] Rasella D, Hone T, de Souza LE, Tasca R, Basu S, Millett C (2019). Mortality associated with alternative primary healthcare policies: a nationwide microsimulation modelling study in Brazil. BMC Med.

[8] Trajman A, Saraceni V, Durovni B, Trajman A, Saraceni V, Durovni B (2018). Sustainable Development Goals and tuberculosis in Brazil: challenges and potentialities. Cad Saúde Pública.

[9] de Souza RA, Nery JS, Rasella D (2018). Family health and conditional cash transfer in Brazil and its effect on tuberculosis mortality. Int J Tuberc Lung Dis.

[10] Pelissari DM, Bartholomay P, Jacobs MG (2018). Offer of primary care services and detection of tuberculosis incidence in Brazil. Rev Saude Publica.

[11] Cidacs Cohort of 100 million Brazilians. https://cidacs.bahia.fiocruz.br/en/platform/cohort-of-100-million-brazilians.

[12] World Without Poverty Unified Registry. http://wwp.org.br/en/social-policy/unified-registry/.

[13] Ali MS, Ichihara MY, Lopes LC (2019). Administrative data linkage in Brazil: potentials for health technology assessment. Front Pharmacol.

[14] Pita R, Pinto C, Sena S (2018). On the accuracy and scalability of probabilistic data linkage over the Brazilian 114 million cohort. IEEE J Biomed Health Inform.

[15] Pinto C, Pita R, Barbosa G, et al. Probabilistic integration of large Brazilian socioeconomic and clinical databases. 2017 IEEE 30th International Symposium on Computer-Based Medical Systems (CBMS), June 22–24, 2017.

[16] Barreto M, Alves A, Sena S, et al. Assessing the accuracy of probabilistic record linkage of social and health databases in the 100 million Brazilian cohort. International Population Data Linkage Conference, 24–26 Aug, 2016.

[17] Pita R, Pinto C, Barreto M (2017). Design and evaluation of probabilistic record linkage methods supporting the Brazilian 100-million cohort initiative. Int J Popul Data Sci.

[18] Pescarini JM, Williamson E, Ichihara MY (2020). Conditional cash transfer program and leprosy incidence: analysis of 12·9 million families from the 100 million Brazilian cohort. Am J Epidemiol.

[19] Pescarini JM, Williamson E, Nery JS (2020). Effect of a conditional cash transfer programme on leprosy treatment adherence and cure in patients from the nationwide 100 Million Brazilian Cohort: a quasi-experimental study. Lancet Infect Dis.

[20] Hone T, Saraceni V, Medina Coeli C (2020). Primary healthcare expansion and mortality in Brazil's urban poor: a cohort analysis of 1·2 million adults. PLoS Med.

[21] Torrens AW, Rasella D, Boccia D (2016). Effectiveness of a conditional cash transfer programme on TB cure rate: a retrospective cohort study in Brazil. Trans R Soc Trop Med Hyg.

[22] Ali MS, Prieto-Alhambra D, Lopes LC (2019). Propensity score methods in health technology assessment: principles, extended applications, and recent advances. Front Pharmacol.

[23] Austin PC, Stuart EA (2015). Moving towards best practice when using inverse probability of treatment weighting (IPTW) using the propensity score to estimate causal treatment effects in observational studies. Stat Med.

[24] Cole SR, Hernán MA (2008). Constructing inverse probability weights for marginal structural models. Am J Epidemiol.

[25] Lee BK, Lessler J, Stuart EA (2011). Weight trimming and propensity score weighting. PLoS One.

[26] Hone T, Macinko J, Millett C (2018). Revisiting Alma-Ata: what is the role of primary health care in achieving the Sustainable Development Goals?. Lancet.

[27] Macinko J, Harris MJ (2015). Brazil's family health strategy—delivering community-based primary care in a universal health system. N Engl J Med.

[28] Departamento de Doenças de Condições Crônicas e Infecções Sexualmente Transmissíveis (2020). Boletim Epidemiológico de Turbeculose. http://www.aids.gov.br/pt-br/pub/2020/boletim-epidemiologico-de-turbeculose-2020.

[29] Aquino R, de Oliveira NF, Barreto ML (2009). Impact of the family health program on infant mortality in Brazilian municipalities. Am J Public Health.

[30] Hone T, Rasella D, Barreto ML, Majeed A, Millett C (2017). Association between expansion of primary healthcare and racial inequalities in mortality amenable to primary care in Brazil: a national longitudinal analysis. PLoS Med.

