# Supplementary material for: The effect of primary health care on tuberculosis in a nationwide cohort of 7·3 million Brazilian people: a quasi-experimental study
Source: Lancet Glob Health. 2022 Jan 24;10(3):e390–7. doi: 10.1016/S2214-109X(21)00550-7 (PMC8847211; doi:10.1016/S2214-109X(21)00550-7)
Supplement: Portuguese translation of the abstract [file mmc1.pdf]

THE LANCET
Global Health

Supplementary appendix 1

This translation in Portuguese was submitted by the authors and we reproduce it as supplied. It has not been peer reviewed. The Lancet's editorial processes have only been applied to the original in English, which should serve as reference for this manuscript.

Supplement to: Jesus GS, Pescarini JM, Silva AF, et al. The effect of primary health care on tuberculosis in a nationwide cohort of 7·3 million Brazilian people: a quasi-experimental study. *Lancet Glob Health* 2022; published online Jan 24. https://doi.org/10.1016/S2214-109X(21)00550-7.

Esta tradução em português foi submetida pelos autores e nós não fizemos quaisquer alterações. Esta versão não foi revista por pares. O processo editorial do The Lancet só foi aplicado à versão original em inglês, que deve servir como referência para este artigo.

Resumo

Antecedentes. A cobertura universal de saúde é uma das intervenções prioritárias da Estratégia pelo Fim da Tuberculose da OMS e pode ser alcançada – particularmente em países de renda baixa e média – por meio da expansão da Atenção Primária à Saúde. Avaliamos os efeitos de um dos maiores programas de atenção primária à saúde do mundo, o Programa Brasileiro de Estratégia de Saúde (ESF), sobre morbidade e mortalidade por tuberculose usando uma coorte nacional de 7,3 milhões de indivíduos ao longo de um período de 10 anos.

Métodos. Analisamos os indivíduos que entraram na Coorte 100 Milhões de Brasileiros durante o período de 1º de janeiro de 2004 até 31/12/2013, e comparamos residentes em municípios sem cobertura da ESF com residentes em municípios com cobertura total da ESF. Usamos um desenho de coorte com regressões multivariáveis de Poisson, ajustadas para todas as variáveis demográficas e socioeconômicas e ponderadas com probabilidade inversa de ponderação do tratamento, para estimar o efeito da ESF na incidência, mortalidade, cura e letalidade da tuberculose. Também realizamos uma série de estratificações e análises de sensibilidade.

Achados. A exposição à ESF foi associada a menor incidência de tuberculose (Razão de Taxas [RR] 0,78; IC 95% 0,72–0,84) e mortalidade (0,72; 0,55–0,94), e foi positivamente associado às taxas de cura da tuberculose (1,04; 1,00–1,08). A ESF também foi associada a uma diminuição nas taxas de letalidade por TB, embora isso não tenha sido estatisticamente significativo (RR 0,84; 95% CI 0,55-1,30). As associações da ESF foram mais fortes entre os mais pobres para todos os indicadores de tuberculose.

Interpretação. Os cuidados primários de saúde, baseados na comunidade, podem reduzir fortemente a morbidade e mortalidade por tuberculose, e diminuir a distribuição desigual da carga da tuberculose nas populações mais vulneráveis. Diante do aumento acentuado da pobreza global devido à pandemia de COVID-19, os investimentos em cuidados primários de saúde podem ajudar a proteger contra os aumentos esperados na incidência de tuberculose e contribuir para o alcance da Estratégia pelo Fim da Tuberculose em todo o mundo.

Financiamento. TB Modeling and Analysis Consortium (Fundação Bill & Melinda Gates), Wellcome Trust e Ministério da Saúde do Brasil.
