# Supplementary material for: The effect of primary health care on tuberculosis in a nationwide cohort of 7·3 million Brazilian people: a quasi-experimental study
Source: Lancet Glob Health. 2022 Jan 24;10(3):e390–7. doi: 10.1016/S2214-109X(21)00550-7 (PMC8847211; doi:10.1016/S2214-109X(21)00550-7)
Supplement: Supplementary appendix 2 [file mmc2.pdf]

THE LANCET
Global Health

Supplementary appendix 2

This appendix formed part of the original submission and has been peer reviewed. We post it as supplied by the authors.

Supplement to: Jesus GS, Pescarini JM, Silva AF, et al. The effect of primary health care on tuberculosis in a nationwide cohort of 7·3 million Brazilian people: a quasi-experimental study. *Lancet Glob Health* 2022; published online Jan 24. https://doi.org/10.1016/S2214-109X(21)00550-7.

Supplementary Webappendix

The Impact of Primary Health Care on Tuberculosis: A Quasi-Experimental Study Based on a Nationwide Cohort of 7.3 Million Brazilians

Table of contents

1.Definitions – Datasets………………………………………………………………..	2
Web Table 1………………………………………………………………………....	3
Web Figure 1………………………………………………………………………..	4
2.Direct Acyclic Graph (DAG) ………………………………………………………..	4
Web Figure 2………………………………………………………………………..	4
3.Descriptive analysis of the subpopulations…………...…………….………………..	5
Web Table 2………………………………………………………………………....	6
4.Estimation of logistic regression for the exposure group, by outcome……………...	8
Web Table 3………………………………………………………………………....	8
5.Stratification for educational levels…………………………………………………	9
Web Table 4………………………………………………………………………..	10
6. Sensitivity Analyses..	11
Web Table 5………………………………………………………………………..	12
Web Table 6………………………………………………………………………..	13
Web Table 7………………………………………………………………………..	14
Web Table 8………………………………………………………………………..	15
Web Table 9………………………………………………………………………..	16
Web Table 10..……………………………………………………………………..	16
Web Table 11..……………………………………………………………………..	17
Web Table 12..……………………………………………………………………..	18
Web Table 13..……………………………………………………………………..	21
7. TB outcomes by age groups………………………………………....................	22
Web Table 14……………………………………………………………………....	22
8. IPTW Poisson regression stratified models…………………………………………	22
Web Table 15………………………………………………………………………..	23
Web Table 16………………………………………………………………………..	24
Web Table 17………………………………………………………………………..	25
Web Table 18………………………………………………………………………..	26
Web Figure 3………………………………………………………………………..	27
Web Figure 4………………………………………………………………………..	28
References…………………………………………………………………………	29

1. Definitions

Datasets

Cadastro Único (CadUnico)

The Cadastro Único (CadUnico) is a national administrative system that contains information about individuals, and their families, that has selectable characteristics to apply for any social programs in Brazil.[1] To be eligible and register with CadUnico, families must receive an income of up to half a minimum wage per capita or a total family income of up to 3 minimum wages (for example, the minimum wage ranged from R $ 380.00 in 2007 to R $ 724.00 in 2014). At the end of 2017, CadUnico consisted of approximately 114 million individual registrations, representing around 50% of the Brazilian population.

At the time of registration, registrants are assigned a unique numerical identifier and researched for socioeconomic indicators. Families must update their records every two years as long as they remain enrolled in any public program. The CadUnico cohort includes 246 socioeconomic and demographic variables with information from the family and individuals registered in this data collection and storage system.[1]

The 100 Million Brazilian Cohort

The Cohort of 100 million Brazilians was created with the objective of enabling studies and continuous assessments of social determinants and the effects of social programs and policies on the different health contexts in Brazil.[2] Created by the Center for Data and Knowledge Integration for Health (CIDACS / FIOCRUZ),[3,4] the cohort is based on information from more than 114 million individuals who were registered during the years 2000 and 2017 in the CadUnico, linked with available health datasets of morbidity and mortality.

In the 100 Million Brazilians Cohort changes of participants' residence are not included and handled in the analysis because information about the date of such changes are not available. However, considering the low rate of internal migration in the country[5] we assume that it has not biased meaningfully our analyses.

In this study, we consider the intervention as the Family Health Strategy (FHS) and the adopted outcomes are: incidence rates, cure rate, case-fatality and mortality rate. New cases of Tuberculosis (TB) are registered in the Notifiable Diseases Information System (SINAN) and the records of deaths as a cause of death TB were collected in the Mortality Information System (SIM).

SINAN includes information on all mandatory reporting diseases in the country. After TB detection, health professionals, both in the public and private sectors, are required to notify the electronic system, reporting the date of clinical detection and demographic characteristics of the patient and updating information about treatment. The SIM provides microdata of deaths by age, sex, cause and municipality of residence of the deceased. The baseline of the cohort constructed by Cidacs / Fiocruz and linked to TB information from SINAN and SIM.

All individuals diagnosed with TB before the beginning of the study period (2004) were excluded from the study cohort. While this could be considered a case of left truncation,1 the inclusion of individuals with a previous event of TB (left censoring) could have biased the analyses, because their tendency to reactivate the TB and become an new incident case would have been greater than individuals without previous TB episodes, and the probability to contract TB before the study period could have been influenced by unknown previous levels of PHC coverage.[6]

Family Health Strategy (FHS)

The FHS was launched in 1994 and has experienced a dramatic expansion across Brazil's municipalities. FHS is currently one of the world's largest community based primary health care programme, involving interdisciplinary health care teams that include a physician, a nurse, a nurse assistant, and four to six full-time community health agents. Family health teams are organized geographically, covering populations of up to 1000 households, and each FHS team member has defined roles and responsibilities. Moreover, national guidelines help to structure and standardize FHS responses to most health problems. Each community health agent is assigned to approximately 150 households in a geographically delineated micro-area within the catchment area — usually the same micro-area where the agent lives. Agents visit each household within their micro-area at least once per month and collect individual- and household-level data. During each visit they develop health promotion activities, also helping scheduling appointments, check whether prescriptions have been filled and whether patients have been taking their medications regularly. They also ask about changes to household composition, identifying signs of violence and neglect, among other problems. They also actively look for risk factors such as smoking and symptoms of common chronic disease such as hypertension and diabetes[7].

FHS teams are located near people's homes, facilitating the access and first-contact care. Moreover, the lists of all residents in each geographic area permit delivery of longitudinal care, and each team is responsible for everyone in its catchment area. The care provided by the FHS model is proactive, since the community health agents seek out problems before patients arrive at the health post.

In Brazil, TB diagnosis and treatment is fully offered - free of charge - by the national health system (Sistema Unico de Saúde - SUS). The TB program was decentralized to primary health care units in 2004, that include both the FHS and other centres that offer the first level of care. The SUS offers screening for latent TB infection, preventive treatment, and TB treatment and all are available free of charge. Although community wide screening for active TB can take place in certain areas from time to time, its not a national recommendation to be adopted by primary health care in routine.

The annual coverage of the FHS is built from information collected from the Ministry of Health, through the Primary Care Information System (SIAB), the most updated and consolidated data on the coverage of the FHS in all 5,570 Brazilian municipalities in the period 2000 to 2018. Here we will present the data from 2004 to 2013 due to the reduction of the study period discussed in the body of the article. The FHS exposure variable was calculated as the total number of teams deployed in the municipality that year multiplied by 3,450, which represents the number of people served by each FHS team, divided by the population of the municipality. CadUnico's registrations were linked to the coverage of the municipal FHS by the municipality code. A summary table of FHS municipal coverage and national coverage is shown on the Web Table 1, and a graph of the annual distributions of FHS municipal coverage (box plot) is shown in Web Figure 1.

Web Table 1. **Average number of Family Health Strategy (FHS) teams and coverage of the health strategy of the municipal and national family 2004 – 2013.**

Year	Municipal		National	
	Team FHS[1]	% FHS Coverage[2]	Team FHS[3]	% FHS Coverage[4]
2000	1.24	25.7	6,835	14.20
2001	2.00	38.6	11,106	22.23
2002	2.78	51.2	15,484	30.59
2003	3.25	57.2	18,043	35.19
2004	3.70	62.4	20,578	39.10
2005	4.12	67.0	22,928	42.95
2006	4.70	73.4	26,130	48.27
2007	4.97	76.2	27,645	51.83
2008	5.17	77.3	28,770	52.35
2009	5.38	78.6	29,961	53.97
2010	5.60	80.6	31,185	56.41
2011	5.78	81.4	32,182	57.71
2012	5.91	81.3	32,906	58.54
2013	6.13	81.4	34,127	58.56

Notes: [1]Average number of municipal FHS teams; [2]Average municipal FHS coverage; [3] Number of FHS teams in the all country; [4] FHS coverage in the country.

Web Figure 1. Box plox of the distributions of FHS municipal coverage from 2000 to 2013.

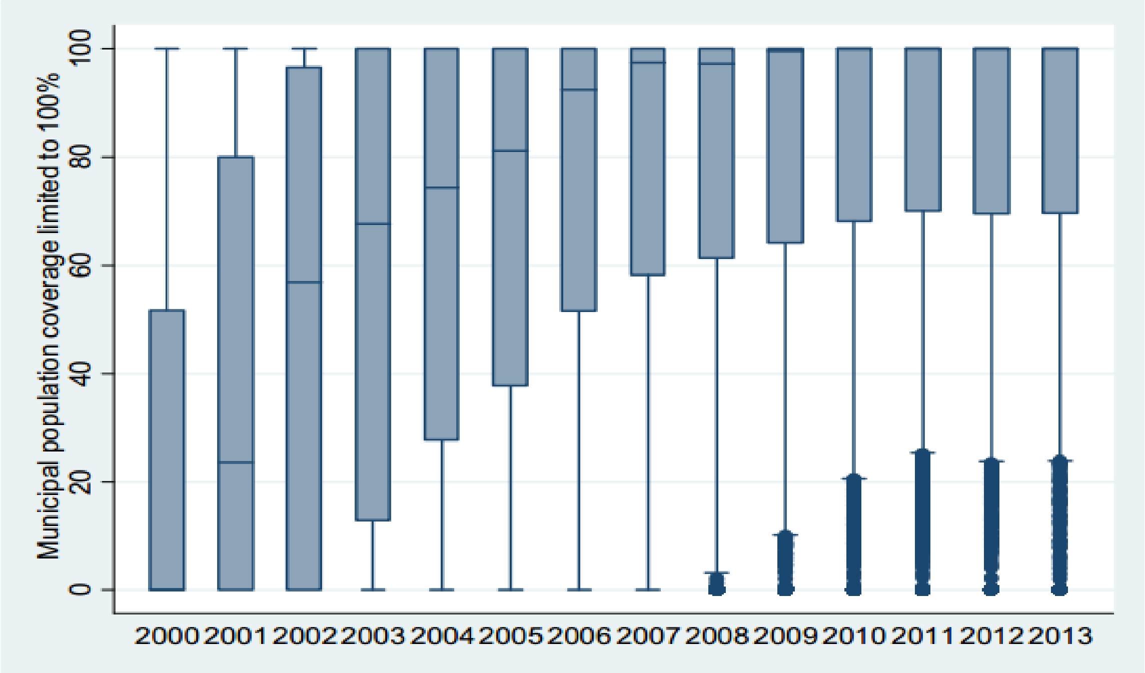

2. Direct Acyclic Graph (DAG)

In order to disentangle the causal relationships and detect possible biases due to the choice of the adjusting variables in the logistic and Poisson multivariable regression models, we created a Direct Acyclic Graph (DAG) of the variables selected through the theoretical framework, the dataset availability, and the better prediction of the region of common support for the propensity score (Web Figure 2).

Web Figure 2. Direct Acyclic Graph (DAG).

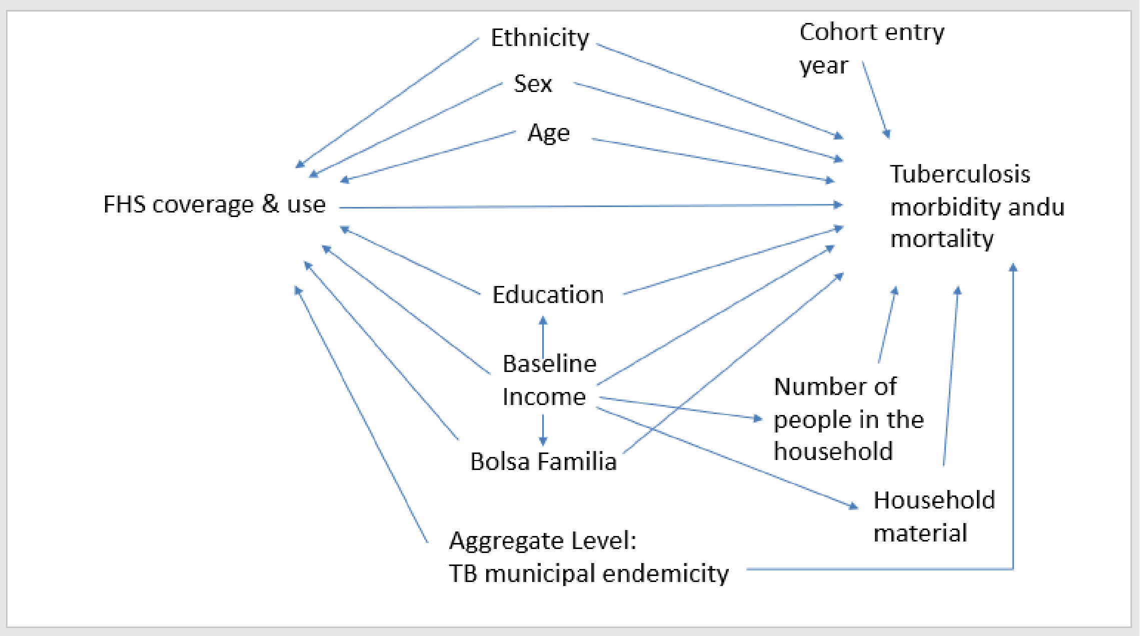

3. Descriptive analysis of the subpopulations

The descriptive analysis of the data by population strata is shown in Web Table 2. The subpopulations are divided into two strata for each variable, and they are described by group exposed to the FHS (<10% and 100%). The income subpopulations were built according to the median per capita family income. Education subpopulations were stratified by individuals who are illiterate or have up to four years of study and individuals who have 5 years or more of schooling. The last two subpopulations were based on sex (male and female) and age (under 15 and 15 years old or more).

Web Table 2. Description of individuals covered by the Family Health Strategy for each subpopulation in the cohort of 100 million Brazilians, Brazil, 2004–2013

	INCOME								EDUCATION							
	Below the median				Above the median				Less 4 years of study				5 years of study or more			
	<10%		100%		<10%		100%		<10%		100%		<10%		100%	
	n	% or SD[1]	n	% or SD	n	% or SD	n	% or SD	n	% or SD	n	% or SD	n	% or SD	n	% or SD
**Age (median)**	18.6	15.52	19.64	16.58	22.36	19.57	22.89	20.76	18.14	21.69	19.96	21.94	25.17	12.48	23.26	10.94
**Sex**																
Men	382,086	52.0	1,886,340	56.7	723,846	46.5	1,505,251	50.9	631,504	50.8	2,221,236	57.0	358,470	44.9	815,978	48.0
Woman	353,302	48.0	1,442,970	43.3	834,263	53.5	1,453,300	49.10	612,395	49.2	1,675,499	43.0	439,118	55.1	882,875	52.0
**Family per capita income[2]**																
Below the median	-	-	-	-	-	-	-	-	370,348	29.8	1,982,228	50.9	252,098	31.6	892,795	52.6
Above the median	-	-	-	-	-	-	-	-	873,551	70.20	1,914,507	49.1	545,490	68.4	806,058	47.4
**Receiving time Bolsa Família (months)**	70.01	39.73	83.75	40.19	46.59	40.57	53.86	44.65	55.17	41.23	70.21	43.88	50.58	41.87	67.19	46.44
**Construction material Household**																
Bricks/cement	581,147	81.4	2,211,298	68.7	1,348,797	87.4	2,204,723	76.2	1,030,141	83.6	2,713,135	70.7	693,987	87.7	1,257,396	75.0
Wood, other vegetal materials	133,012	18.6	1,008,308	31.3	193,570	12.6	687,557	23.8	202,592	16.4	1,124,525	29.3	97,098	12.3	418,256	25.0
**Education[3]**																
Illiterate	179,571	28.8	954,276	33.2	436,701	30.8	1,053,752	38.7	-	-	-	-	-	-	-	-
Primary school or less	190,777	30.6	1,027,952	35.8	436,850	30.8	860,755	31.6	-	-	-	-	-	-	-	-
Junior high school	187,440	30.1	688,605	24.0	339,499	23.9	507,424	18.7	-	-	-	-	-	-	-	-
High school	64,658	10.4	204,190	7.1	205,991	14.5	298,634	11.0	-	-	-	-	-	-	-	-
**Race**																
White	385,258	55.6	852,832	27.5	854,297	57.5	993,990	35.1	689,978	56.4	1,121,331	29.5	435,522	57.4	552,227	34.1
Black	45,656	6.6	207,098	6.7	85,244	5.7	167,338	5.9	67,498	5.50	241,778	6.4	49,884	6.6	100,379	6.2
Brown	262,569	37.9	2,046,843	65.9	547,427	36.8	1,669,312	59.0	465,598	38.1	2,436,044	64.1	273,904	36.1	968,598	59.7
**Number of people in the family**																
≤ 2	82,969	11.3	478,857	14.4	321,719	20.6	749,377	25.3	224,514	18.0	749,367	19.2	134,821	16.9	328,655	19.3
2< e ≤4	323,070	43.9	1,512,578	45.4	767,221	49.2	1,438,571	48.6	582,930	46.9	1,830,805	47.0	389,381	48.8	806,355	47.5
≥5	329,349	44.8	1,337,875	40.2	469,169	30.1	770,603	26.0	436,455	35.1	1,316,563	33.8	273,386	34.3	563,843	33.2

Table continues

Continued Web Table 2. Description of individuals covered by the Family Health Strategy for each subpopulation in the cohort of 100 million Brazilians, Brazil, 2004–2013.

	SEX								AGE							
	Men				Women				Less than 15 years				15 years or more			
	<10%		100%		<10%		100%		<10%		100%		<10%		100%	
	n	% or SD[1]	n	% or SD	n	% or SD	n	% or SD	n	% or SD	n	% or SD	n	% or SD	n	% or SD
**Age (median)**	20.71	18.59	22.58	18.79	21.58	18.32	19.51	18.52	-	-	-	-	-	-	-	-
**Sex**																
Men	-	-	-	-	-	-	-	-	551,116	51.0	1,384,607	51.2	554,816	45.7	2,006,984	56.0
Woman	-	-	-	-	-	-	-	-	529,378	49.0	1,322,198	48.8	658,187	54.3	1,574,072	44.0
**Family per capita income[2]**																
Below the median	382,086	34.5	1,886,340	55.6	353.302	29,8	1,442,970	49.8	37,245	34.5	1,447,996	53.5	362,937	29.9	1,881,314	52.5
Above the median	723,846	65.5	1,505,251	44.4	834.263	70.2	1,453,300	50.2	708,043	65.5	1,258,809	46.5	850,066	70.1	1,699,742	47.5
**Receiving time Bolsa Família (months)**	53.53	41.5	69.89	44.7	54.64	41.98	69.45	45.03	60.49	39.45	73.81	40.51	48.41	42.92	66.57	47.71
**Construction material Household**																
Bricks/cement	924,725	85.1	2,361,401	71.5	1.005.219	86.0	2,054,620	73.1	895,455	84.1	1,887,910	71.9	1,034,489	86.8	2,528,111	72.5
Wood, other vegetal materials	162,297	14.9	941,658	28.5	164,285	14.0	754,207	26.9	168,729	15.9	738,691	28.1	157,853	13.2	957,174	27.5
**Education[3]**																
Illiterate	312,738	31.6	1,108,332	36.5	303.534	28.9	899,696	35.2	531,160	52.7	1,473,624	58.3	85,112	8.2	534,404	17.4
Primary school or less	318,766	32.2	1,112,904	36.6	308.861	29.4	775,803	30.3	293,700	29.1	737,057	29.2	333,927	32.3	1,151,650	37.5
Junior high school	251,461	25.4	607,688	20.0	275.478	26.2	588,341	23.0	179,348	17.8	309,346	2.2	347,591	33.7	886,683	28.9
High school	107,009	10.8	208,290	6.9	163.640	15.6	294,534	11.5	4,619	0.5	8,428	0.3	266,030	25.8	494,396	16.1
**Race**																
White	595,133	56.7	965,402	30.3	644.422	57.0	881,420	32.0	599,726	56.4	802,422	30.6	639,829	57.3	1,044,400	31.5
Black	62,177	5.9	209,071	6.6	68.723	6.10	165,365	6.0	47,718	4.5	120,404	4.6	83,182	7.5	254,032	7.7
Brown	392,084	37.4	2,011,183	63.1	417.912	36.9	1,704,972	62.0	416,655	39.2	1,696,951	64.8	393,341	35.2	2,019,204	60.9
**Number of people in the family**																
≤ 2	143,165	12.9	531,147	15.7	261.523	22.0	697,087	24.1	116,595	10.8	329,818	12.2	288,093	23.8	898,416	25.1
2< e ≤4	534,319	48.3	1,634,676	48.2	555.972	46.8	1,316,473	45.5	530,389	49.1	136,102	50.3	559,902	46.2	1,590,126	44.4
≥5	428,448	38.7	1,225,768	36.1	370.070	31.2	882,710	30.5	433,510	40.1	1,015,964	37.5	365,008	30.1	1,092,514	30.5

[1]SD: Standard Deviation

[2]Family per capita income: median per capita income

[3]Primary school or less: ≤5 years of education; junior high school: 6–9 years of education; high school: ≥10 years of education.

4. Estimation of logistic regression for the exposure group, by outcome

For each dataset of the outcome variable, we estimate the probability of the individual being exposed to the FHS, that is, residing in a municipality that has 100% coverage of primary health care using multivariable logistic regression adjusted for the relevant demographic and socioeconomic covariables (Web Table 3). Covariables include sex, age of entry into the cohort, education, race, number of people in the family, household supplies, family income per capita, time spent receiving the conditional cash transfer policy, Bolsa Família, average municipal TB incidence, AIDS and diabetes and year of entry into the cohort.

Web Table 3. Prediction models using logistic regression for the individual to live in municipalities with FHS coverage (<10% and 100%) in the 100 Million Brazilian Cohort for cutouts with TB outcomes.

	Incidence & Mortality		Cure rate		Fatality rate	
	**OR[1]**	**95% CI[2]**	**OR**	**95% CI**	**OR**	**95% CI**
Woman	0.88	0.88 - 0.88	0.87	0.72 - 1.06	0.82	0.71 - 0.95
Age	1.00	1.00 - 1.00	1.00	1.00 - 1.01	1.01	1.00 - 1.01
Edc[3]: <5 years	0.70	0.70 - 0.70	0.44	0.35 - 0.58	0.44	0.36 - 0.54
Edc: ≥ 5 e ≤ 9 years	0.57	0.56 - 0.57	0.28	0.22 - 0.39	0.31	0.25 - 0.38
Edc: ≥ 10 years	0.63	0.63 - 0.64	0.27	0.19 - 0.39	0.30	0.23 - 0.40
Eth[4]: Black	2.25	2.23 - 2.27	2.05	1.56 - 2.71	1.98	1.58 - 2.47
Eth: Brown	3.51	3.50 - 3.53	3.44	2.86 - 4.16	3.02	2.60 - 3.51
Num/fam[5]: 2< e ≤ 4	0.70	0.69 - 0.70	0.52	0.40 - 0.67	0.56	0.46 - 0.68
Num/fam: ≥ 5	0.50	0.50 - 0.51	0.37	0.28 - 0.48	0.40	0.32 - 0.49
House/material[6]: Others (wood, other vegetal materials)	1.70	1.69 - 1.70	2.03	1.64 - 2.52	2.39	2.02 - 2.83
Income[7]: Above the median	0.53	0.53 - 0.54	0.55	0.46 - 0.66	0.54	0.47 - 0.62
BFP[8] (months)	1.00	1.00 - 1.00	1.004	1.00 - 1.01	1.00	1.00 - 1.01
Munic. Incidence	0.98	0.98 - 0.98	0.98	0.97 - 0.99	0.98	0.98 - 0.98
AIDS	-	-	2.08	1.44 - 3.02	1.42	1.08 - 1.86
Diabetes	-	-	1.06	0.71 - 1.59	0.95	0.69 - 1.30
Year of registry in the cohort						
2005	1.38	1.36 - 1.39	1.40	0.97 - 2.02	1.34	0.98 - 1.82
2006	2.39	2.37 - 2.41	2.77	2.06 - 3.74	2.57	2.01 - 3.28
2007	1.99	1.97 - 2.01	2.53	1.79 - 3.58	2.54	1.92 - 3.36
2008	1.48	1.46 - 1.49	1.64	1.04 - 2.59	1.54	1.07 - 2.27
2009	1.44	1.43 - 1.46	1.36	0.75 - 2.44	1.22	0.80 - 1.86
2010	1.00	0.99 - 1.01	0.73	0.39 - 1.35	1.07	0.70 - 1.63
2011	1.35	1.34 - 1.37	0.72	0.24 - 2.13	1.17	0.79 - 1.89
2012	1.76	1.74 - 1.78	-	-	1.48	0.91 - 2.41
2013	1.63	1.61 - 1.65	-	-	0.44	0.11 - 1.72
**Obs.:**	7,308,968		3,379		5,368	

Notes: [1]Oddis Ratio; [2] Confidence Interval; [3]Edc, Education; [4]Eth, Ethnicity; [5] Num/fam, Number of people in the family; [6]House/material, Houselhold material; [7] Income, Family per capita income; [8]BFP, Bolsa Familia Program. Reference variable: Sex: Men; Education: Never attended; Ethnicity/race: White; Person by domicile: ≤ 2; Houselhold material: Brick; Family per capita income: Below the median (0.5 BR$/month); Year of registry in the cohort: 2004.

5. Stratification for educational levels

Web Table 4 presents the RRs for the education subpopulations, divided in less than 4 years of education and 5 years or more, for each TB outcome.

Web Table 4. Rate Ratio for Incidence, Mortality, Cure and Fatality Rates due to Tuberculosis by coverage of the Family Health Strategy (FHS) for education subpopulation, in the study cohort (Brazil, 2004– 2013). Poisson weighted with IPTW.

	**Incidence**				**Mortality**				**Cure rate**				**Fatality rate**			
	**Less 4y**		**5 y or more**		**Less 4y**		**5y or more**		**Less 4y**		**5y or more**		**Less 4y**		**5y or more**	
	**RR[1]**	**95% IC[2]**	**RR**	**95% IC**	**RR**	**95% IC**	**RR**	**95% IC**	**RR**	**95% IC**	**RR**	**95% IC**	**RR**	**95% IC**	**RR**	**95% IC**
**FHS 100%**	0.78	(0.71 - 0.87)	0.73	(0.65 - 0.81)	0.72	(0.53 - 0.97)	0.78	(0.46 - 1.30)	0,99	(0.94-1.05)	1,07	(1.03-1.12)	0,91	(0.57-1.46)	1,13	(0.54-2.34)
Woman	0.56	(0.51 - 0.61)	0.63	(0.56 - 0.69)	0.40	(0.30 - 0.53)	0.39	(0.24 - 0.64)	1,01	(0.95-1.08)	1,08	(1.03-1.12)	0,60	(0.35-1.02)	0,49	(0.23-1.03)
Age	1.43	(1.41 - 1.45)	1.30	(1.25 - 1.35)	1.57	(1.50 - 1.65)	1.82	(1.64 - 2.01)	0,99	(0.97-1.00)	0,98	(0.96-1.00)	1,24	(1.10-1.38)	0,45	(1.10-1.89)
Eth[3]: Black	1.90	(1.66 - 2.18)	1.69	(1.41 - 2.03)	2.74	(1.90 - 3.95)	2.70	(1.30 - 5.57)	1,01	(0.93-1.09)	0,89	(0.80-0.97)	1,76	(1.00-3.08)	0,87	(0.30-2.48)
Eth: Brown	1.38	(1.25 - 1.51)	1.16	(1.04 - 1.30)	2.08	(1.60 - 2.69)	2.04	(1.12 - 3.73)	1,03	(0.97-1.09)	1,01	(0.97-1.05)	1,79	(1.08- 2.91)	1,05	(0.50-2.18)
Num/fam[4]: 2< e ≤ 4	0.83	(0.75 - 0.93)	0.79	(0.68 - 0.93)	0.78	(0.58 - 1.04)	0.70	(0.38 - 1.30)	1,04	(0.96-1.12)	1,07	(1.00-1.16)	0,59	(0.34-1.00)	0,61	(0.22-1.64)
Num/fam: ≥ 5	0.93	(0.83 - 1.04)	0.99	(0.84 - 1.17)	0.96	(0.68 - 1.34)	0.88	(0.43 - 1.77)	1,04	(0.96-1.13)	1,07	(0.99-1.14)	0,88	(0.52-1.49)	1,19	(0.43-3.26)
House/material[5]: Others (wood, other vegetal materials)	1.03	(0.94 - 1.12)	1.15	(1.02 - 1.31)	1.22	(0.95 - 1.57)	1,32	(0.77 - 2.27)	0,99	(0.93-1.05)	1,01	(0.95-1.06)	0,87	(0.54-1.38)	2,90	(1.37-6.12)
Income6: Above the median	0.86	(0.79 - 0.93)	0.93	(0.84 - 1.04)	0.88	(0.67 - 1.16)	0.63	(0.42 - 0.95)	0,97	(0.92-1.02)	1,02	(0.97-1.06)	1,03	(0.63-1.67)	0,36	(0.19-0.66)
BFP7 (months)	1.00	(1.00 - 1.00)	1.00	(1.00 - 1.00)	0.99	(0.99 - 1.00)	0.99	(0.99 - 1.00)	1,00	(1.00-1.00)	1,00	(0.99-1.00)	0,99	(0.99-1.00)	0,99	(0.98-1.00)
Munic. Incidence	1.00	(1.00 - 1.00)	1.00	(1.00 - 1.00)	1.00	(1.00 - 1.00)	1.00	(1.00 - 1.00)	0,99	(0.99-1.00)	1,00	(1.00-1.00)	0,99	(0.99-1.00)	0,99	(0.99-1.00)
AIDS	-	-	-	-	-	-	-	-	1,72	(1.37-2.16)	1,50	(1.23-1.82)	5,58	(0.77-40.43)	9,57	(1.19-76.77)
Diabetes	-	-	-	-	-	-	-	-	0,96	(0.87-1.06)	0,92	(0.82-1.02)	0,85	(0.44-1.65)	3,19	(0.38-26.81)
**Obs.:**	4,954,813		2,354,155		4,954,813		2,354,155		1.802		1.577		2.870		2.498	

Abbreviations: [1] RR, Rate Ratios; [2] CI, Confidence Interval; [3]Eth, Ethnicity; [4] Num/fam, Number of people in the family; [5] House/material, Houselhold material; [6] Income, Family per capita income; [7] BFP, Bolsa Familia Program.

Foot note: Reference variable: Sex: Men; Ethnicity/race: White; Person by domicile: ≤ 2; Houselhold material: Brick; Family per capita income: Below the median (0.5 BR$/month).

6. Sensitivity Analyses

We performed several sensitivity analyses.

First, while - in order to have a more equitable ratio between exposed and not exposed individuals - we used in the main analyses individuals in municipalities with <10% FHP coverage as not exposed, we tested if using 0% FHS coverage was affecting the results. Web Table 5 presents the results for FHS 0% and 100% coverage, showing FHS effect estimates similar in direction, magnitude and statistical significance to the one presented as main results, with the exception of cure rate, probably due to the reduced number of observations (2,067).

Second, to evaluate the robustness of our results, we used a different quasi-experimental approach, the Propensity Score Matching, estimating the FHS impact Average Treatment on the Treated (ATT) (Web Table 6). All ATT measure were comparable in direction, magnitude and statistical significance with the main results of the study, except for cure rate probably due to the same reason explained above.

Third, to verify the relevance of the IPTW for unbiased FHS estimates, we run the same multivariable Poisson regressions without IPTW and compared with the ones with IPTW (Web Table 7). All FHS impact estimates were comparable in direction, magnitude and statistical significance with the main results of the study, however the FHS effects were considerably stronger on TB incidence and mortality without IPTW correction.

Fourth, to test the relevance of the variable that adjust for the endemic levels of TB in each municipality, we estimated the same regressions without the TB cumulative incidence as adjusting variable (Web Table 8). As above, all FHS impact estimates were comparable in direction, magnitude and statistical significance with the main results of the study, however the FHS effects were considerably stronger on TB incidence and mortality without adjusting for the TB average annual incidence.

Fifth, to verify the relevance of the adjustment for all covariates specifically in the Poisson regression, we run the models as Poisson bivariate regression (without IPTW) between the TB outcome and the FHS exposure (Web Table 7), even if we adjusted the previous logistic regression for PS with all relevant variables: the adjustment at the Poisson level, even if in the majority of the case is not changing the direction and statistical significance of the association, is an important factor of adjustment for the effects magnitude.

Sixth, to test the relevance of including other aggregated covariates, we have included the most TB-relevant municipal level variables available in our datasets (Web Table 10). These include: a. municipal Gini index – representing the level of social inequalities, b. municipal percentage of sanitation coverage – representing sanitation and other municipal infrastructures, and c. municipal density of nurses – representing the level of coverage of healthcare professionals most relevant for the detection and follow-up of TB patients. The introduction of all these aggregate-level variables in the models did not meaningfully change the FHS estimates of effectiveness. We also tested the municipal Intraclass Correlation Coefficient (ICC) for TB incidence and mortality rates (Web Table 9): 0.005 and 0.0004 respectively, and an ICC close to 0 – from a pure statistical point of view -do not justify the use of a multilevel analysis.

Seventh, we have evaluated to stratify the analysis based on changes of diagnostic/treatment. No major point-of-care diagnostic innovations have been implemented in Brazil during the study period, considering the Gene Xpert diagnostic was nationally implemented in only at the end of 2013, corresponding also to the end of our study period. However, we have stratified the analyses on TB cure rates according to the introduction of the four-drug fixed-dose combination regimen in 2009 (Web Table 13), showing no important differences (the loss of significance of the >=2009 is possibly due to the reduced number of TB cases – n.667).

Eight, in order to be sure that such exclusion was not affecting FHP impact estimates, we recoded the missing values of each categorical variable with a specific code for missing: considering they were included in the regression models as dummy variables, and the code for missing was not used as baseline category for the dummy estimation, this approach did not change the effect of each covariate. For continuous variables (age in years and months of Bolsa Familia receiving), we imputed the missing values as the median of their distribution. We successively fitted the same regression models of the study using all 8,581,358 individuals, obtaining FHP effect estimates (together with other covariate estimates) almost identical to main results presented in the manuscript, with an FHS RR 0.79 (95%CI: 0.74-0.85) versus the original RR 0.78 (95%CI: 0.72-0.84) (Web Table 11). We also compared the descriptive analyses of each variable of the 8,581,358 individuals versus the 7,308,968 used in the main analyses, and we found no meaningful differences (Web Table 12).

Web Table 5. Rate ratio for Incidence, Mortality, Cure and Case-Fatality rates due to Tuberculosis by Family Health Strategy coverage (0 and 100%), in the study cohort (Brazil, 2004– 2013). Poisson weighted with IPTW.

	Incidence		Mortality		Cure rate		Fatality rate	
**Poisson adjusted**	**RR[1]**	**95% IC[2]**	**RR**	**95% IC**	**RR**	**95% IC**	**RR**	**95% IC**
FHS 100%	0.70	(0.63-0.78)	0.88	(0.60-1.29)	0.93	(0.88-0.98)	0.94	(0.58-1.52)
Woman	0.61	(0.57-0.65)	0.42	(0.32-0.54)	1.01	(0.96-1.06)	0.63	(0.39-1.02)
Age	1.46	(1.44-1.48)	1.69	(1.62-1.76)	0.99	(0.97-1.00)	1.32	(1.17-1.50)
Edc[3]: <5 years	1.27	(1.17-1.37)	0.78	(0.62-0.99)	1.07	(0.99-1.14)	0.88	(0.57-1.37)
Edc: ≥ 5 e ≤ 9 years	1.65	(1.51-1.80)	0.78	(0.57-1.06)	1.07	(0.99-1.14)	0.88	(0.47-1.64)
Edc: ≥ 10 years	1.623	(1.42-1.86)	0.39	(0.20-0.72)	1.06	(0.96-1.17)	0.59	(0.21-1.64)
Eth[4]: Black	1.74	(1.55-1.95)	2.37	(1.61-3.47)	0.91	(0.82-1.00)	1.77	(1.00-3.12)
Eth: Brown	1.34	(1.24-1.44)	1.87	(1.41-2.46)	1.02	(0.96-1.07)	1.46	(0.21-1.64)
Num/fam[5]: 2< e ≤ 4	0.75	(0.68-0.82)	0.75	(0.56-1.00)	1.03	(0.95-1.10)	0.77	(0.47-1.26)
Num/fam: ≥ 5	0.86	(0.77-0.94)	0.86	(0.63-1.16)	1.01	(0.93-1.09)	1.01	(0.60-1.70)
House/material[6]: Others (wood, other vegetal materials)	1.02	(0.95-1.10)	1.04	(0.83-1.29)	1.00	(0.95-1.05)	1.14	(0.74-1.66)
Income[7]: Above the median	0.89	(0.83-0.96)	0.89	(0.70-1.11)	0.99	(0.94-1.04)	0.82	(0.55-1.22)
BFP[8] (months)	1.00	(1.00-1.00)	0.99	(0.99-1.00)	1.00	(0.99-1.00)	0.99	(0.99-1.00)
Munic. Incidence	1.00	(1.00-1.00)	1.00	(1.00-1.00)	1.00	(0.99-1.00)	1.00	(0.99-1.00)
AIDS	-	-	-	-	1.68	(1.35-2.10)	3.49	(0.85-14.34)
Diabete	-	-	-	-	1.00	(0.89-1.13)	0.81	(0.43-1.51)
**Year of registry in the cohort**								
2005	1.01	(0.87-1.16)	0.88	(0.56-1.38)	1.01	(0.90-1.11)	0.92	(0.38-2.24)
2006	1.08	(0.96-1.21)	0.90	(0.62-1.30)	0.98	(0.90-1.06)	0.89	(0.43-1.81)
2007	1.13	(0.99-1.30)	1.04	(0.68-1.58)	0.97	(0.88-1.07)	0.68	(0.29-1.55)
2008	1.07	(0.88-1.29)	1.23	(0.70-2.13)	0.95	(0.82-1.10)	0.86	(0.31-2.37)
2009	0.85	(0.68-1.06)	0.29	(0.11-0.80)	1.11	(0.96-1.27)	0.38	(0.09-1.54)
2010	1.33	(1.03-1.69)	1.07	(0.43-2.62)	0.75	(0.54-1.04)	0.31	(0.06-1.51)
2011	0.87	(0.62-1.22)	1.47	(0.66-3.28)	1.11	(0.83-1.48)	0.73	(0.20-2.60)
2012	1.00	(0.72-1.39)	1.82	(0.83-3.98)	-	-	1.75	(0.62-4.95)
2013	0.86	(0.39-1.90)	2.98	(0.71-12.48)	-	-	5.06E-06	(1.59E-06-0.00)
**Obs.:**	5,314,209		5,314,209		2,067		3,387	

Abbreviations: [1] RR, Rate ratios; [2] CI, Confidence Interval; [3] Edc, Education; [4] Eth, Ethnicity; [5] Num/fam, Number of people in the family; [6] House/material, Houselhold material; [7] Income, Family per capita income; [8]BFP, Bolsa Familia Program.

Foot note: Reference variable: Sex: Men; Education: Never attended; Ethnicity/race: White; Person by domicile: ≤ 2; Houselhold material: Brick; Family per capita income: Below the median (0.5 BR$/month); Year of registry in the cohort: 2004.

Web Table 6. Average effect of treatment of FHS coverage of <10% and 100% on incidence and mortality (in number of cases / deaths over 100,000 inhabitants) and cure and lethality (in percentage) from tuberculosis in the study cohort (Brazil, 2004– 2013).

	Incidence	Mortality	Cure rate	Fatality rate
ATT treated[1]	77.78	7.39	84.63	4.01
ATT control	111.60	13.38	87.34	4.91
ATT diff[2]	-33.82*	-5.99*	-2.71	-0.89
Observations	6,343,608	6,343,608	3,379	5,368

Foot note: [1]Average Treatment on the Treated; [2] Difference between group treated and control; [3] Statistic T: *p-value <0,05.

Web Table 7. Rate ratio for Incidence, Mortality, Cure and Case-Fatality rates due to Tuberculosis by Family Health Strategy (FHS) coverage, in the study cohort (Brazil, 2004– 2013). Results of the Poisson regression without IPTW weights, not adjusted and adjusted.

	Incidence		Mortality		Cure rate		Fatality rate	
**Poisson not adjusted**	**RR[1]**	**95% IC[2]**	**RR**	**95% IC**	**RR**	**95% IC**	**RR**	**95% IC**
**FHS 100%**	0.44	0.43 - 0.46	0.73	0.62 - 0.86	1.01	0.95 - 1.07	1.40	1.07 - 1.82
**Obs.:**	8,581,358		8,581,358		5,407		8,123	
**Poisson adjusted**	**RR**	**95% IC**	**RR**	**95% IC**	**RR**	**95% IC**	**RR**	**95% IC**
FHS 100%	0.60	0.57 - 0.64	0.68	0.55 - 0.85	1.03	0.94 - 1.12	0.87	0.60 - 1.27
Woman	0.56	0.53 - 0.59	0.40	0.33 - 0.48	1.02	0.94 - 1.11	0.71	0.49 - 1.04
Age	1.40	1.38 - 1.41	1.67	1.60 - 1.73	0.98	0.96 - 1.01	1.35	1.22 - 1.49
Edc[3]: <5 years	1.33	1.24 - 1.41	0.88	0.73 - 1.05	1.01	0.90 - 1.13	1.05	0.72 - 1.53
Edc: ≥ 5 e ≤ 9 years	1.87	1.75 - 2.00	0.80	0.63 - 1.02	1.03	0.92 - 1.16	0.73	0.44 - 1.21
Edc: ≥ 10 years	1.87	1.70 - 2.04	0.62	0.41 - 0.94	1.08	0.93 - 1.26	0.60	0.27 - 1.36
Eth[4]: Black	1.90	1.75 - 2.05	2.44	1.85 - 3.21	0.97	0.86 - 1.11	1.65	1.03 - 2.65
Eth: BrownBrown	1.38	1.31 - 1.46	1.90	1.56 - 2.31	1.02	0.94 - 1.11	1.24	0.86 - 1.78
Num/fam[5]: 2< e ≤ 4	0.82	0.76 - 0.87	0.74	0.60 - 0.92	1.03	0.92 - 1.15	0.73	0.48 - 1.11
Num/fam: ≥ 5	0.95	0.89 - 1.03	0.81	0.65 - 1.03	1.03	0.92 - 1.16	0.90	0.58 - 1.39
House/material[6]: Others (wood, other vegetal materials)	1.07	1.01 - 1.12	1.20	1.00 - 1.43	0.98	0.89 - 1.07	1.12	0.78 - 1.59
Income[7]: Above the median	0.87	0.82 - 0.91	0.76	0.64 - 0.91	1.00	0.92 - 1.08	0.69	0.49 - 0.96
BFP[8] (months)	1.00	1.00 - 1.00	0.99	0.99 - 1.00	1.00	0.99 - 1.00	0.99	0.99 - 1.00
Munic. Incidence	1.00	1.00 - 1.00	1.00	1.00 - 1.00	1.00	0.99 - 1.00	0.99	0.99 - 0.99
AIDS	-	-	-	-	1.65	1.35 - 2.01	5.08	1.25 - 20.58
Diabetes	-	-	-	-	0.93	0.78 - 1.12	1.03	0.59 - 1.81
Year of registry in the cohort								
2005	0.89	0.81 - 0.98	0.98	0.70 - 1.36	1.02	0.87 - 1.20	1.25	0.62 - 2.50
2006	0.98	0.91 - 1.07	0.89	0.68 -1.18	1.01	0.88 - 1.15	1.22	0.68 - 2.18
2007	0.98	0.89 - 1.09	1.02	0.74 - 1.41	1.01	0.87 - 1.19	0.97	0.49 - 1.91
2008	0.93	0.81 - 1.05	0.96	0.64 - 1.44	1.04	0.85 - 1.28	0.86	0.36 - 2.06
2009	0.81	0.70 - 0.94	0.58	0.33 - 0.99	1.04	0.80 - 1.35	0.65	0.21 - 2.01
2010	1.04	0.90 - 1.21	0.88	0.52 - 1.48	0.98	0.74 - 1.30	0.57	0.18 - 1.77
2011	0.82	0.68 - 0.98	0.79	0.45 - 1.39	1.09	0.68 - 1.74	1.04	0.36 - 2.93
2012	0.94	0.79 - 1.12	1.27	0.79 - 2.03	-	-	2.19	0.94 - 5.09
2013	0.66	0.42 - 1.05	3.14	1.60 - 6.16	-	-	6.10e-06	0 - .
Intercept	0.00	0.00 - 0.00	0.00	0.00 - 0.00	0.50	0.34 - 0.72	0.00	0.00 - 0.02
**Obs.:**	9,308,968		9,308,968		3,379		5,368	

Notes: [1]Rate ratios; [2] Confidence Interval; [3]Edc, Education; [4]Eth, Ethnicity; [5] Num/fam, Number of people in the family; [6]House/material, Houselhold material; [7] Income, Family per capita income; [8]BFP, Bolsa Familia Program.

Foot note: Reference variable: Sex: Men; Education: Never attended; Ethnicity/race: White; Person by domicile: ≤ 2; Houselhold material: Brick; Family per capita income: Below the median (0.5 BR$/month); Year of registry in the cohort: 2004.

Web Table 8. Rate ratio for Incidence, Mortality, Cure and Fatality rates due to Tuberculosis by Family Health Strategy coverage (<10% and 100%), in the study cohort (Brazil, 2004– 2013). Poisson weighted with IPTW without adjusting for the municipal TB incidence variable.

	Incidence		Mortality		Cure rate		Fatality rate	
**Poisson adjusted**	**RR[1]**	**95% IC[2]**	**RR**	**95% IC**	**RR**	**95% IC**	**RR**	**95% IC**
FHS 100%	0.35	(0.33-0.37)	0.45	(0.36-0.56)	1.02	(0.99-1.06)	0.94	(0.67-1.33)
Woman	0.61	(0.58-0.65)	0.41	(0.32-0.52)	1.03	(0.99-1.07)	0.60	(0.39-0.93)
Age	1.42	(1.41-1.44)	1.67	(1.60-1.74)	0.98	(0.97-1.06)	1.30	(1.15-1.46)
Edc[3]: <5 years	1.45	(1.35-1.57)	0.96	(0.77-1.20)	1.01	(0.95-1.07)	0.97	(0.62-1.51)
Edc: ≥ 5 e ≤ 9 years	2.11	(1.95-2.28)	1.02	(0.77-1.35)	1.04	(0.98-1.10)	0.68	(0.37-1.25)
Edc: ≥ 10 years	2.16	(1.94-2.41)	0.62	(0.38-1.03)	1.07	(1.01-1.14)	0.59	(0.24-1.46)
Eth[4]: Black	2.08	(1.90-2.28)	2.64	(1.90-3.66)	0.97	(0.91-1.03)	1.51	(0.93-2.45)
Eth: Brown	1.47	(1.39-1.57)	2.37	(1.86-3.01)	1.01	(0.98-1.05)	1.41	(0.96-2.06)
Num/fam[5]: 2< e ≤ 4	0.79	(0.73-0.86)	0.67	(0.51-0.88)	1.04	(0.98-1.09)	0.58	(0.37-0.92)
Num/fam: ≥ 5	0.93	(0.85-1.01)	0.75	(0.56-1.00)	1.03	(0.97-1.09)	0.87	(0.53-1.40)
House/material[6]: Others(wood, other vegetal materials)	1.08	(1.01-1.15)	1.23	(0.99-1.53)	1.00	(0.96-1.04)	1.30	(0.88-1.92)
Income[7]: Above the median	0.88	(0.83-0.94)	0.82	(0.67-1.01)	0.99	(0.95-1.02)	0.72	(0.88-1.92)
BFP[8] (months)	1.00	(1.00-1.00)	0.99	(0.66-7.18)	1.00	(0.99-1.00)	0.99	(0.99-1.00)
AIDS	-	-	-	-	1.72	(1.45-2.03)	7.52	(1.84-30.71)
Diabete	-	-	-	-	0.96	(0.88-1.05)	0.82	(0.45-1.48)
**Year of registry in the cohort**								
2005	0.92	(0.82-1.03)	1.07	(0.72-1.59)	1.03	(0.96-1.11)	0.96	(0.45-2.08)
2006	1.07	(0.98-1.18)	0.92	(0.65-1.29)	1.01	(0.95-1.07)	0.90	(0.46-1.77)
2007	1.00	(0.89-1.12)	1.05	(0.71-1.55)	1.03	(0.96-1.10)	0.78	(0.35-1.74)
2008	0.88	(0.76-1.03)	0.90	(0.54-1.51)	1.06	(0.97-1.16)	0.68	(0.25-1.78)
2009	0.77	(0.65-0.92)	0.46	(0.20-1.05)	1.02	(0.91-1.14)	0.36	(0.11-1.20)
2010	1.07	(0.89-1.29)	0.94	(0.47-1.87)	0.92	(0.77-1.10)	0.53	(0.15-1.87)
2011	0.80	(0.62-1.03)	1.00	(0.44-2.26)	1.12	(0.99-1.00)	0.67	(0.23-1.98)
2012	0.87	(0.67-1.14)	1.78	(0.90-3.52)	-	-	1.41	(0.56-3.56)
2013	0.57	(0.29-1.11)	2.18	(0.66-7.18)	-	-	8.69E-07	(2.76E-07-2.73E-06)
**Obs.:**	6,343,608		6,343,608		3,379		5,368	

Abbreviations: [1] RR, Rate ratios; [2] CI, Confidence Interval; [3] Edc, Education; [4] Eth, Ethnicity; [5] Num/fam, Number of people in the family; [6] House/material, Houselhold material; [7] Income, Family per capita income; [8]BFP, Bolsa Familia Program.

Foot note: Reference variable: Sex: Men; Education: Never attended; Ethnicity/race: White; Person by domicile: ≤ 2; Houselhold material: Brick; Family per capita income: Below the median (0.5 BR$/month); Year of registry in the cohort: 2004.

Web Table 9. Mixed-effects Poisson regression

	INCIDENCE	MORTALITY
**Obs.:**	8.581.358	8.581.358
**Groups**	1.384	1.384
**Var**	0,3512959	0,321925
**X'B**	-9,146029	-11,41124
**ICC**	0,005350282	0,000493853

Web Table 10. IPTW Poisson regression models, adjusted for all demographic, socioeconomic and ecological variables, for the association between tuberculosis outcomes and the Family Health Strategy (FHS) coverage in the study cohort (Brazil, 2004– 2013).

	Incidence		Mortality	
	RR[1]	95% IC[2]	RR	95% IC
FHS 100%	0.77	0.71 -0.83	0.72	0.54 - 0.95
Woman	0.59	0.55 - 0.63	0.39	0.31 - 0.51
Age	1.41	1.39 - 1.43	1.59	1.52 - 1.67
Edc[3]: <5 years	1.3	1.19 - 1.41	0.94	0.74 - 1.19
Edc: ≥ 5 e ≤ 9 years	1.79	1.64 - 1.96	0.79	0.57 - 1.09
Edc: ≥ 10 years	1.84	1.63 - 2.09	0.48	0.29 - 0.78
Eth[4]: Black	1.82	1.63 - 2.03	2.59	1.82 - 3.68
Eth: Brown	1.29	1.02 - 1.39	1.91	1.49 - 2.46
Num/fam[5]: 2< e ≤ 4	0.82	0.75 - 0.90	0.73	0.56 - 0.96
Num/fam: ≥ 5	0.96	0.88 - 1.06	0.93	0.68 - 1.28
House/material[6]: Others (wood, other vegetal materials)	1.1	1.02 - 1.18	1.08	0.86 - 1.35
Income[7]: Above the median	1.88	0.82 - 0.94	0.84	0.66 - 1.09
BFP[8] (months)	1,00	1.00 - 1.00	0.99	0.99 - 1.00
Munic. Incidence	1,00	1.00 - 1.00	1,00	1.00 - 1.00
Percentage of household with sewage	0.99	0.99 - 1.00	1,00	0.99 - 1.01
Gini Index	1,00	0.99 - 1.00	1.01	0.99 - 1.03
Number of Nurses per 1,000 inhabitants	1.21	1.09 - 1.34	0.83	0.57 - 1.21
**Obs.:**	7,276,017			

Abbreviations: [1]RR, Rate ratios; [2]CI, Confidence Interval; [3]Edc, Education; [4]Eth, Ethnicity; [5] Num/fam, Number of people in the family; [6]House/material, Houselhold material; [7] Income, Family per capita income; [8]BFP, Bolsa Familia Program.
Foot note: Reference variable: Sex: Men; Education: Never attended; Ethnicity/race: White; Person by domicile: ≤ 2; Houselhold material: Brick; Family per capita income: Below the median (0.5 BR$/month).

Web Table 11. IPTW Poisson regression models, adjusted for all demographic and socioeconomic variables, including missings, for the association between Tuberculosis Incidence and the Family Health Strategy (FHS), 2004-2008 and 2009-2013 (Brazil).

	Incidence	
	**RR[1]**	**95% IC[2]**
FHS 100%	0.79	(0.74-0.85)
Woman	0.59	(0.55-0.63)
Age	1.39	(1.37-1.40)
Edc[3]: <5 years	1.29	(1.18-1.40)
Edc: ≥ 5 e ≤ 9 years	1.74	(1.59-1.90)
Edc: ≥ 10 years	1.81	(1.60-2.04)
Missing	1.39	(1.24-1.56)
Eth[4]: Black	1.81	(1.63-2.01)
Eth: Brown	1.30	(1.21-1.39)
Missing	1.47	(1.27-1.70)
Num/fam[5]: <2 e ≤ 4	0.86	(0.79-0.93)
Num/fam: ≥ 5	0.99	(0.91-1.09)
House/material[6]: Others (wood, other vegetal materials)	1.09	(1.02-1.17)
Missing	1.42	(1.15-1.75)
Income[7]: Above the median	0.89	(0.83-0.95)
BFP[8] (months)	1.00	(1.00-1.00)
Munic. Incidence	1.00	(1.00-1.00)
**Obs.:**	8,581,358	

Abbreviations: [1]RR, Rate ratios; [2]CI, Confidence Interval; [3]Edc, Education; [4]Eth, Ethnicity; [5] Num/fam, Number of people in the family; [6]House/material, Houselhold material; [7] Income, Family per capita income; [8]BFP, Bolsa Familia Program.

Foot note: Reference variable: Sex: Men; Education: Never attended; Ethnicity/race: White; Person by domicile: ≤ 2; Houselhold material: Brick; Family per capita income: Below the median (0.5 BR$/month).

Web Table 12. Descriptions of missings and non-missings.

	No- missing	Missing	Total
**Sex**			
Man	3.850.625	646.898	4,497,523
	85,62	14,38	100.00
	52,68	50,84	52.41
Woman	3,458,343	625.492	4,083,835
	84.68	15,32	100.00
	47.32	49.16	47.59
**Total**	**7,308,968**	**1,272,390**	**8,581,358**
	85.17	14.83	100.00
	100.00	100.00	100.00
**Education**			
Never attended	2,541,119	83.181	2,624,300
	96.83	3.17	100.00
	34.77	25.35	34.36
Primary school or less (<5 years of education)	2,413,694	102.640	2,516,334
	95.92	4.08	100.00
	33.02	31.28	32.95
Junior high school (≥ 5 e ≤ 9 years of education)	1,648,733	74,235	1,722,968
	95.69	4.31	100.00
	22.56	22.63	22.56
High school (≥ 10 years of education)	705,422	68,051	773,473
	91.20	8.80	100.00
	9.65	20.74	10.13
**Total**	**7,308,968**	**328,107**	**7,637,075**
	95.70	4.30	100.00
	100.00	100.00	100.00
**Ethnicity/race**			
White	2,766,200	320,177	320,177
	89.63	10.37	100.00
	37.85	39.58	38.02
Black	454,418	50,918	505,336
	89.92	10.08	100.00
	6.22	6.29	6.22
Brown	4,088,350	437,801	4,526,151
	90.33	9.67	100.00
	55.94	54.12	55.76
**Total**	**7,308,968**	**808,896**	**8,117,864**
	90.04	9.96	100.00
	100.00	100.00	100.00
**Number of people in the family**			
≤ 2	1,345,088	287,834	1,632,922
	82.37	17.63	100.00

	18.40	22.62	19.03
2< e ≤4	3,467,289	574,151	4,041,440
	85.79	14.21	100.00
	47.44	45.12	47.10
≥ 5	2,496,591	410,405	2,906,996
	85.88	14.12	100.00
	34.16	32.25	33.88
**Total**	**7,308,968**	**1,272,390**	**8,581,358**
	85.17	14.83	100.00
	100.00	100.00	100.00
**House/material**			
Brick/cement	5,528,220	817,745	6,345,965
	87.11	12.89	100.00
	75.64	77.19	75.83
Others (wood, other vegetal materials)	1,780,748	241,699	2,022,447
	88.05	11.95	100.00
	24.36	22.81	24.17
**Total**	**7,308,968**	**1,059,444**	**8,368,412**
	87.34	12.66	100.00
	100.00	100.00	100.00
**Family per capita income**			
Bellow the median (0.5 BR$/month)	3,377,775	686,923	4,064,698
	83.10	16.90	100.00
	46.21	53.99	47.37
Above the median (0.5 BR$/month)	3,931,193	585,467	4,516,660
	87.04	12.96	100.00
	53.79	46.01	52.63
**Total**	**7,308,968**	**1,272,390**	**8,581,358**
	85.17	14.83	100.00
	100.00	100.00	100.00
**Year of registry in the cohort**			
2004	477,932	94,328	572,26
	83.52	16.48	100.00
	6.54	7.41	100.00
2005	530,605	114,878	645,483
	82.20	17.80	100.00
	7.26	9.03	7.52
2006	2,622,965	548,234	3,171,199
	82.71	17.29	100.00
	35.89	43.09	36.95
2007	928,724	138,144	1,066,868
	87.05	12.95	100.00
	12.71	10.86	12.43
2008	437,344	70,108	507,452

	86.18	13.82	100.00
	5.98	5.51	5.91
2009	466,369	55,144	521,513
	89.43	10.57	100.00
	6.38	4.33	6.08
2010	406,747	38,088	444,835
	91.44	8.56	100.00
	5.57	8.56	5.18
2011	429,182	25,286	454,468
	94.44	5.56	100.00
	5.87	1.99	5.30
2012	592,502	47,488	639,99
	92.58	7.42	100.00
	8.11	3.73	7.46
2013	416,598	140,692	557,29
	74.75	25.25	100.00
	5.70	11.06	6.49
**Total**	**7,308,968**	**1,272,390**	**8,581,358**
	85.17	14.83	100.00
	100.00	100.00	100.00

Web Table 13. IPTW Poisson regression models, adjusted for all demographic and socioeconomic variables, for the association between Cure Rate and Case-fatality rate Tuberculosis and the Family Health Strategy (FHS), 2004-2008 and 2009-2013 (Brazil).

	**Cure rate**			
	**2004-2008**		**2009-2011**	
	**RR[1]**	**95% IC[2]**	**RR**	**95% IC**
FHS 100%	1.06	(1.02-1.10)	1.03	(0.92-1.15)
Woman	1.02	(1.00-1.07)	1.06	(0.94-1.19)
Age	0.99	(0.97-1.00)	0.98	(0.94-1.02)
Edc[3]: <5 years	1.00	(0.95-1.06)	1.13	(0.97-1.32)
Edc: ≥ 5 e ≤ 9 years	1.04	(0.98-1.11)	1.10	(0.94-1.29)
Edc: ≥ 10 years	1.09	(1.02-1.17)	1.08	(0.89-1.31)
Eth[4]: Black	0.98	(0.92-1.03)	0.95	(0.75-1.21)
Eth: Brown	1.00	(0.97-1.04)	1.04	(0.92-1.17)
Num/fam[5]: 2< e ≤ 4	1.01	(0.96-1.06)	1.19	(1.02-1.39)
Num/fam: ≥ 5	1.00	(0.95-1.05)	1.10	(0.93-1.32)
House/material[6]: Others (wood, other vegetal materials)	0.99	(0.95-1.03)	1.09	(0.94-1.26)
Income[7]: Above the median	0.98	(0.95-1.02)	0.90	(0.79-1.03)
BFP[8] (months)	1.00	(0.99-1.00)	1.00	(0.99-1.00)
Munic. Incidence	1.00	(0.99-1.00)	0.99	(0.99-1.00)
AIDS	1.73	(1.50-1.99)	1.61	(1.13-2.29)
Diabetes	1.01	(0.92-1.10)	0.87	(0.72-1.05)
**Obs.:**	4,562		617	

Abbreviations: [1]RR, Rate ratios; [2]CI, Confidence Interval; [3]Edc, Education; [4]Eth, Ethnicity; [5] Num/fam, Number of people in the family; [6]House/material, Houselhold material; [7] Income, Family per capita income; [8]BFP, Bolsa Familia Program.

Foot note: Reference variable: Sex: Men; Education: Never attended; Ethnicity/race: White; Person by domicile: ≤ 2; Houselhold material: Brick; Family per capita income: Below the median (0.5 BR$/month).

7. Tuberculosis outcomes per age groups

The 100 Million Brazilian Cohort has a different demographic structure in comparison with the Brazilian population: with 47% of children below 15 years of age (against a national 21%). This implies different overall indicators of TB in comparison with the Brazilian population. While for TB incidence the values of the >15years are compatible with the national TB incidence (37.5 per 100,000 person-years in 2010), TB mortality rates are lower than the national values (2.5 per 100,000 person-years in 2010) because of the underrepresentation in the cohort of the older people.

Web Table 14: Tuberculosis outcomes according to age groups and coverage.

TB Indicators	Age Groups & FHP Coverage			
	<15 years		>15 years	
	<10%	100%	<10%	100%
Incidence Rate (per 100,000 p-y)	8.1	2.7	42.0	18.6
Mortality Rate (per 100,000 p-y)	0.05	0.03	1.2	0.7
Cure Rate (Percentage)	88.3	91.1	81.3	84.2
Case-Fatality Rate (Percentage)	0.66	0.74	2.8	4.4

8. IPTW Poisson regression stratified models

Web Tables 15-18 are showing all the IPTW Poisson regression models, adjusted for all demographic and socioeconomic variables, for the association between Tuberculosis Incidence, Mortality, Cure, Case-fatality rates and the Family Health Strategy (FHS) coverage, overall and according to stratum of income, sex and age (Brazil, 2004– 2013).

Web Table 15. IPTW Poisson regression models, adjusted for all demographic and socioeconomic variables, for the association between Tuberculosis Incidence and the Family Health Strategy (FHS) coverage according to stratum of income, sex and age (Brazil, 2004– 2013).

	General		INCOME				SEX				AGE			
			Below the median		Above the median		Man		Woman		Less than 15 years		15 years or more	
	RR[1]	95% IC[2]	RR	95% IC	RR	95% IC	RR	95% IC	RR	95% IC	RR	95% IC	RR	95% IC
FHS 100%	0.78	0.72 -0.84	0.72	(0.65 - 0.81)	0.85	(0.77 - 0.94)	0.77	(0.70 - 0.84)	0.78	(0.69 - 0.89)	0.64	(0.51 - 0.80)	0.71	(0.66 - 0.77)
Woman	0.59	0.55 - 0.63	0.61	(0.56 - 0.66)	0.58	(0.53 - 0.64)	-	-	-	-	1.02	(0.85 - 1.23)	0.52	(0.49 - 0.56)
Age	1.41	1.39 -1.43	1.44	(1.42 - 1.47)	1.38	(1.35 - 1.41)	1.44	(1.41 - 1.46)	1.34	(1.30 - 1.38)	-	-	-	-
Edc[3]: <5 years	1.30	1.19 - 1.41	1.32	(1.19 - 1.47)	1.28	(1.13 - 1.46)	1.30	(1.18 - 1.44)	1.26	(1.07 - 1.49)	2.25	(1.78 - 2.85)	0.78	(0.72 - 0.86)
Edc: ≥ 5 e ≤ 9 years	1.80	1.64 - 1.96	1.69	(1.51 - 1.89)	1.91	(1.67 - 2.18)	1.76	(1.58 - 1.96)	1.82	(1.54 - 2.15)	3.63	(2.79 - 4.71)	0.70	(0.64 - 0.78)
Edc: ≥ 10 years	1.85	1.64 - 2.10	1.85	(1.57 - 2.18)	1.86	(1.57 - 2.22)	1.67	(1.42 - 1.96)	2.08	(1.69 - 2.54)	3.40	(1.36 - 8.47)	0.57	(0.50 - 0.65)
Eth[4]: Black	1.83	1.64 - 2.04	1.84	(1.59 - 2.12)	1.80	(1.54 - 2.11)	1.77	(1.55 - 2.02)	1.93	(1.60 - 2.33)	1.64	(1.15 - 2.35)	1.68	(1.50 - 1.88)
Eth:Brown	1.29	1.20 - 1.39	1.31	(1.18 - 1.44)	1.32	(1.19 - 1.46)	1.25	(1.15 - 1.37)	1.38	(1.21 - 1.57)	1.45	(1.18 - 1.78)	1.21	(1.12 - 1.30)
Num/fam[5]: 2< e ≤ 4	0.82	0.75 - 0.90	0.75	(0.67 - 0.85)	0.84	(0.74 - 0.950	0.78	(0.70 - 0.88)	0.86	(0.74 - 1.00)	1.43	(1.02 - 2.01)	0.74	(0.68 - 0.81)
Num/fam: ≥ 5	0.96	0.87 - 1.06	0.82	(0.72 - 0.92)	1.10	(0.96 - 1.26)	0.96	(0.85 - 1.08)	0.91	(0.77 - 1.07)	1.85	(1.31 - 2.61)	0.84	(0.76 - 0.93)
House/material[6]: Others (wood, other vegetal materials)	1.08	1.01 - 1.16	1.02	(0.93 - 1.11)	1.16	(1.04 - 1.30)	1.05	(0.96 - 1.15)	1.15	(1.01 - 1.30)	1.18	(0.95 - 1.48)	1.00	(0.93 - 1.08)
Income[7]: Above the median	0.88	0.83 – 0.95	-	-	-	-	0.87	(0.80 – 0.95)	0.92	(0.82 – 1.03)	0.81	(0.67 - 0.99)	0.96	(0.89 – 1.03)
BFP[8] (months)	1.00	1.001 - 1.003	1.00	(1.00 – 1.00)	1.00	(1.00 – 1.00)	1.00	(1.00 – 1.00)	1.00	(1.00 – 1.00)	1.00	(1.00 - 1.00)	1.00	(1.00 – 1.00)
Munic. Incidence	1.00	1.004 - 1.005	1.00	(1.00 – 1.00)	1.00	(1.00 – 1.00)	1.00	(1.00 – 1.00)	1.00	(1.00 – 1.00)	1.00	(1.00 - 1.00)	1.00	(1.00 – 1.00)
**Obs.:**	7,308,968		3,377,775		3,931,193		3,850,625		3,458,343		3,436,592		3,872,376	

Abbreviations: [1]RR, Rate ratios; [2]CI, Confidence Interval; [3]Edc, Education; [4]Eth, Ethnicity; [5] Num/fam, Number of people in the family; [6]House/material, Houselhold material; [7] Income, Family per capita income; [8]BFP, Bolsa Familia Program.

Foot note: Reference variable: Sex: Men; Education: Never attended; Ethnicity/race: White; Person by domicile: ≤ 2; Houselhold material: Brick; Family per capita income: Below the median (0.5 BR$/month).

Web Table 16. IPTW Poisson regression models, adjusted for all demographic and socioeconomic variables, for the association between Tuberculosis Mortality and the Family Health Strategy (FHS) coverage according to stratum of income, sex and age (Brazil, 2004– 2013).

Poisson adjusted	General		INCOME				SEX				AGE			
			Below the median		Above the median		Man		Woman		Less than 15 years		15 years or more	
	RR[1]	95% IC[2]	RR	95% IC	RR	95% IC	RR	95% IC	RR	95% IC	RR	95% IC	RR	95% IC
FHS 100%	0.72	0.55 - 0.94	0.71	(0.47 - 1.06)	0.76	(0.54 - 1.06)	0.66	(0.49 - 0.90)	0.87	(0.50 - 1.49)	0.30	(0.11 - 0.78)	0.73	(0.55 - 0.96)
Woman	0.40	0.31 - 0.51	0.41	(0.29 - 0.58)	0.40	(0.29 - 0.56)	-	-	-	-	0.73	(0.27 - 1.96)	0.37	(0.28 - 0.47)
Age	1.59	1.51 - 1.67	1.68	(1.59 - 1.78)	1.56	(1.45 - 1.68)	1.65	(1.55 - 1.75)	1.45	(1.34 - 1.50)	-	-	-	-
Edc[3]: <5 years	0.91	0.72 - 1.16	0.95	(0.71 -1.29)	0.89	(0.63 - 1.26)	1.03	(0.78 - 1.35)	0.57	(0.36 - 0.91)	1.24	(0.38 - 3.98)	0.56	(0.44 - 0.71)
Edc: ≥ 5 e ≤ 9 years	0.76	0.55 - 1.06	0.89	(0.59 - 1.33)	0.65	(0.39 - 1.08)	0.86	(0.60 -1.24)	0.52	(0.26 - 1.05)	0.71	(0.19 - 2.56)	0.26	(0.19 - 0.36)
Edc: ≥ 10 years	0.46	0.29 - 0.75	0.48	(0.23 - 1.01)	0.48	(0.25 - 0.90)	0.40	(0.22 - 0.73)	0.46	(0.21 - 1.01)	0.00	(0.00 - 0.00)	0.11	(0.07 - 0.18)
Eth[4]: Black	2.74	1.94 - 3.89	2.92	(1.81 - 4.70)	2.37	(1.49 - 3.77)	2.43	(1.60 - 3.69)	3.62	(2.05 - 6.38)	10.00	(2.61 - 38.26)	2.30	(1.60 - 3.30)
Eth: Brown	2.09	1.62 - 2.69	1.78	(1.26 - 2.51)	2.34	(1.69 - 3.24)	2.00	(1.49 - 2.67)	2.33	(1.44 - 3.79)	3.27	(1.05 - 10.09)	1.88	(1.45 - 2.43)
Num/fam[5]: 2< e ≤ 4	0.73	0.56 - 0.96	0.78	(0.54 - 1.12)	0.62	(0.42 - 0.91)	0.80	(0.59 - 1.09)	0.50	(0.29 - 0.85)	0.23	(0.05 - 0.98)	0.64	(0.49 - 0.83)
Num/fam: ≥ 5	0.93	0.68 - 1.28	0.80	(0.55 - 1.14)	1.09	(.68 - 1.76)	0.95	(0.66 - 1.38)	0.81	(0.49 - 1.34)	0.53	(0.14 - 2.05)	0.79	(0.58 - 1.07)
House/material[6]: Others (wood, other vegetal materials)	1.20	0.95 - 1.53	1.03	(0.77 - 1.38)	1.46	(1.02 - 2.09)	1.12	(0.85 - 1.48)	1.43	(0.92 - 2.22)	1.64	(0.52 - 5.18)	1.08	(0.85 - 1.36)
Income[7]: Above the median	0.83	0.65 - 1.06	-	-	-	-	0.78	(0.59 - 1.05)	0.99	(0.65 - 1.52)	1.03	(0.34 - 3.04)	0.93	(0.73 - 1.19)
BFP[8] (months)	0.99	0.99 - 1.00	0.99	(0.99 - 0.99)	1.00	(1.00 - 1.00)	0.99	(0.99 - 1.00)	0.99	(0.99 - 1.00)	0.99	(0.98 - 1.01)	0.99	(0.99 - 0.99)
Munic. Incidence	1.00	1.00 - 1.00	1.00	(1.00 - 1.00)	1.00	(1.00 - 1.00)	1.00	(1.00 - 1.00)	1.00	(1.00 - 1.00)	1.00	(0.99 - 1.00)	1.00	(0.99 - 1.00)
**Obs.:**	7,308,968		3,377,775		3,931,193		3,850,625		3,458,343		3,436,592		3,872,376	

Abbreviations: [1]RR, Rate ratios; [2]CI, Confidence Interval; [3]Edc, Education; [4]Eth, Ethnicity; [5] Num/fam, Number of people in the family; [6]House/material, Houselhold material; [7] Income, Family per capita income; [8]BFP, Bolsa Familia Program.

Foot note: Reference variable: Sex: Men; Education: Never attended; Ethnicity/race: White; Person by domicile: ≤ 2; Houselhold material: Brick; Family per capita income: Below the median (0.5 BR$/month).

Web Table 17. IPTW Poisson regression models, adjusted for all demographic and socioeconomic variables, for the association between Tuberculosis Cure Rate and the Family Health Strategy (FHS) coverage according to stratum of income, sex and age (Brazil, 2004– 2013).

**Poisson adjusted**	**General**		**INCOME**				**SEX**				**AGE**			
			**Below the median**		**Above the median**		**Man**		**Woman**		**Less than 15 years**		**15 years or more**	
	**RR[1]**	**95% IC[2]**	**RR**	**95% IC**	**RR**	**95% IC**	**RR**	**95% IC**	**RR**	**95% IC**	**RR**	**95% IC**	**RR**	**95% IC**
FHS 100%	1.04	1.00 - 1.08	1.06	(1.00 - 1.12)	1.02	(0.97 - 1.07)	1.05	(0.99 - 1.10)	1.03	(0.97 - 1.08)	1.01	(0.92 - 1.11)	1.04	(1.00 - 1.08)
Woman	1.03	1.00 - 1.08	1.06	(1.00 - 1.12)	1.02	(0.97 - 1.07)	-	-	-	-	1.08	(0.97 - 1.19)	1.03	(0.98 - 1.07)
Age	0.99	0.97 - 1.00	0.99	(0.97 - 1.00)	0.98	(0.97 - 1.00)	0.99	(0.97 - 1.00)	0.98	(0.96 - 1.00)	-	-	-	-
Edc[3]: <5 years	1.01	0.95 - 1.07	0.99	(0.92 - 1.06)	1.03	(0.94 - 1.13)	0.96	(0.89 - 1.04)	1.07	(0.96 - 1.19)	0.89	(0.80 - 1.01)	1.03	(0.96 - 1.10)
Edc: ≥ 5 e ≤ 9 years	1.03	0.97 - 1.09	0.98	(0.91 - 1.05)	1.10	(1.01 - 1.20)	0.97	(0.89 - 1.05)	1.11	(0.99 - 1.25)	0 .95	(0.87 - 1.03)	1.06	(0.99 - 1.13)
Edc: ≥ 10 years	1.08	1.00 - 1.15	1.12	(1.04 - 1.20)	1.08	(0.97 - 1.20)	1.03	(0.93 - 1.15)	1.14	(1.00 - 1.29)	1.02	(0.92 - 1.14)	1.11	(1.03 - 1.21)
Eth[4]: Black	0.95	0.89 - 1.02	0.94	(0.86 - 1.03)	0.98	(0.89 - 1.07)	0.97	(0.88 - 1.06)	0.89	(0.81 - 0.98)	0.95	(0.78 - 1.17)	0.96	(0.89 - 1.03)
Eth: Brown	1.01	0.98 - 1.05	1.00	(0.94 - 1.05)	1.03	(0.98 - 1.08)	1.02	(0.97 - 1.07)	1.00	(0.95 - 1.05)	1.04	(0.96 - 1.12)	1.01	(0.97 - 1.06)
Num/fam[5]: 2< e ≤ 4	1.06	1.00 - 1.12	1.11	(1.01 - 1.21)	1.03	(0.95 - 1.10)	1.07	(0.98 - 1.17)	1.04	(0.97 - 1.11)	0.88	(0.81 - 0.95)	1.07	(1.01 - 1.13)
Num/fam: ≥ 5	1.05	0.99 - 1.11	1.11	(1.02 - 1.20)	1.01	(0.93 - 1.09)	1.08	(0.98 - 1.18)	1.01	(0.94 - 1.07)	0.85	(0.78 - 0.94)	1.07	(1.00 - 1.13)
House/material[6]: Others (wood, other vegetal materials)	0.99	0.95 - 1.04	0.98	(0.91 - 1.04)	0.98	(0.92 - 1.04)	0.98	(0.92 - 1.04)	1.03	(0.97 - 1.09)	0.93	(0.80 - 1.08)	1.00	(0.95 - 1.05)
Income[7]: Above the median	0.99	0.95 - 1.02	-	-	-	-	1.00	(0.95 - 1.05)	0.99	(0.93 - 1.04)	0.97	(0.89 - 1.04)	0.90	(0.94 - 1.02)
BFP[8] (months)	1.00	0.99 - 1.00	1.00	(0.99 - 1.00)	1.00	(0.99 - 1.00)	1.00	(0.99 - 1.00)	1.00	(0.99 - 1.00)	0.99	(0.99 - 1.00)	1.00	(1.00 - 1.00)
Munic. Incidence	1.00	0.99 - 1.00	1.00	(0.99 - 1.00)	0.99	(0.99 - 1.00)	1.00	(0.99 - 1.00)	0.99	(0.99 - 1.00)	0.99	(0.99 - 1.00)	1.00	(0.99 - 1.00)
AIDS	1.57	1.35 - 1.84	1.51	(1.22 - 1.86)	1.65	(1.30 - 2.03)	1.67	(1.35 - 2.05)	1.47	(1.18 - 1.83)	1.47	(0.92 - 2.34)	1.56	(1.33 - 1.83)
Diabetes	0.94	0.87 - 1.02	0.92	(0.83 - 1.00)	0.97	(0.86 - 1.08)	1.00	(0.88 - 1.12)	0.92	(0.83 - 1.02)	0.87	(0.74 - 1.02)	0.95	(0.87 - 1.03)
**Obs.:**	3,379		1,650		1,729		2,175		1,204		386		2,991	

Abbreviations: [1]RR, Rate ratios; [2]CI, Confidence Interval; [3]Edc, Education; [4]Eth, Ethnicity; [5] Num/fam, Number of people in the family; [6]House/material, Houselhold material; [7] Income, Family per capita income; [8]BFP, Bolsa Familia Program.

Foot note: Reference variable: Sex: Men; Education: Never attended; Ethnicity/race: White; Person by domicile: ≤ 2; Houselhold material: Brick; Family per capita income: Below the median (0.5 BR$/month).

Web Table 18. IPTW Poisson regression models, adjusted for all demographic and socioeconomic variables, for the association between Tuberculosis Case-fatality rate and the Family Health Strategy (FHS) coverage according to stratum of income, sex and age (Brazil, 2004– 2013).

Poisson adjusted	General		INCOME				SEX				AGE			
			Below the median		Above the median		Man		Woman		Less than 15 years		15 years or more	
	RR[1]	95% IC[2]	RR	95% IC	RR	95% IC	RR	95% IC	RR	95% IC	RR	95% IC	RR	95% IC
FHS 100%	0.84	0.55 - 1.30	0.82	(0.44- 1.51)	0.97	(0.53-1.74)	0.81	(0.49-1.31)	0.93	(0.39-2.20)	0.15	(0.01-1.33)	0.92	(0.60-1.42)
Woman	0.60	0.35 - 1.01	0.74	(0.34-1.59)	0.54	(0.26-1.05)	-	-	-	-	0.48	(0.20-1.11)	0.56	(0.34-0.92)
Age	1.19	1.04 - 1.37	1.27	(1.00-1.62)	1.11	(0.97-1.27)	1.26	(1.10-1.45)	1.09	(0.86-1.37)	-	-	-	-
Edc[3]: <5 years	0.66	0.37 - 1.18	1.49	(0.48-4.60)	0.38	(0.19-0.71)	0.74	(0.38-0.41)	0.43	(0.12-1.51)	0.00	(0.00-0.14)	0.64	(0.38-1.07)
Edc: ≥ 5 e ≤ 9 years	0.46	0.20 - 1.04	1.53	(0.33-6.93)	0.10	(0.03-0.28)	0.59	(0.24-1.44)	0.17	(0.03-0.76)	0.29	(0.07-1.16)	0.34	(0.17-0.65)
Edc: ≥ 10 years	0.32	0.11 - 0.92	0.36	(0.04-3.30)	0.27	(0.09-0.76)	0.30	(0.09-0.96)	0.20	(0.03-1.06)	-	-	0.22	(0.08-0.54)
Eth[4]: Black	1.46	0.86 - 2.48	1.64	(0.74-3.64)	1.20	(0.60-2.37)	1.20	(0.64-2.21)	3.48	(1.32-9.12)	-	-	1.46	(0.85-2.50)
Eth: Brown	1.54	1.01 - 2.36	1.47	(0.78-2.76)	1.41	(0.80-2.46)	1.49	(0.93-2.38)	2.37	(1.09-5.15)	1,7e+06	(2,6e+05 - 1.0e+07)	1.45	(0.93-2.26)
Num/fam[5]: 2< e ≤ 4	0.50	0.29 - 0.84	0.70	(0.38-1.27)	0 .36	(0.17-0.72)	0.46	(0.25-0.83)	0.45	(0.16-1.25)	2,8e+04	(1,7e+03 - 4,4e+05)	0.51	(0.31-0.85)
Num/fam: ≥ 5	0.88	0.52 - 1.49	1.15	(0.59-2.22)	0.73	(0.36-1.50)	0.70	(0.37-1.30)	1.85	(0.78-4.37)	1,2e+05	(8,1e+03 - 1,9e+06)	0.83	(0.49-1.41)
House/material[6]: Others (wood, other vegetal materials)	1.18	0.75 - 1.86	1.46	(0.76-2.79)	0.98	(0.49-1.95)	1.25	(0.78-2.01)	0.75	(0.23-2.40)	5.49	(1.29 - 23.22)	1.21	(0.77-1.88)
Income[7]: Above the median	0.84	0.55 - 1.29	-	-	-	-	0.90	(0.55-1.46)	0.84	(0.35-1.96)	-	-	0.99	(0.64-1.50)
BFP[8] (months)	0.99	0.99 - 1.00	0.99	(0.99-1.00)	0.99	(0.99-.00)	0.99	(0.99-1.00)	0.99	(0.98-1.00)	0.99	(0.96- 1.03)	0.99	(0.99-1.00)
Munic. Incidence	0.99	0.99 - 1.00	0.99	(0.99-1.00)	0.99	(0.99-1.00)	0.99	(0.99- 1.00)	0.99	(0.99-1.00)	0.98	(0.96-0.99)	0.99	(0.99-1.00)
AIDS	7.82	1.88 - 32.4	8.99	(1.17-68.88)	7.43	(1.01-54.61)	6.11	(1.45-5.60)	1,5e+06	(8,4e+05 - 2,9e+06)	4,81e+05	(0.43 -1,0e+04)	8.69	(2.11-5.77)
Diabetes	0.98	0.53 - 1.81	0.97	(0.39-2.44)	0.78	(0.37-1.61)	1.03	(0.47-2.23)	0.69	(0.20-2.31)	67.31	(0.43 - 1,0e+04)	0.88	(0.48-1.59)
**Obs.:**	5.368		2,505		2,861		3,520		1,848		727		4,640	

Abbreviations: [1]RR, Rate ratios; [2]CI, Confidence Interval; [3]Edc, Education; [4]Eth, Ethnicity; [5] Num/fam, Number of people in the family; [6]House/material, Houselhold material; [7] Income, Family per capita income; [8]BFP, Bolsa Familia Program.

Foot note: Reference variable: Sex: Men; Education: Never attended; Ethnicity/race: White; Person by domicile: ≤ 2; Household material: Brick; Family per capita income: Below the median (0.5 BR$/month).

Web Figure 3. Cumulative incidence of tuberculosis by percentage of FHS coverage (<10% and 100%) in the study cohort (Brazil, 2004 - 2013).

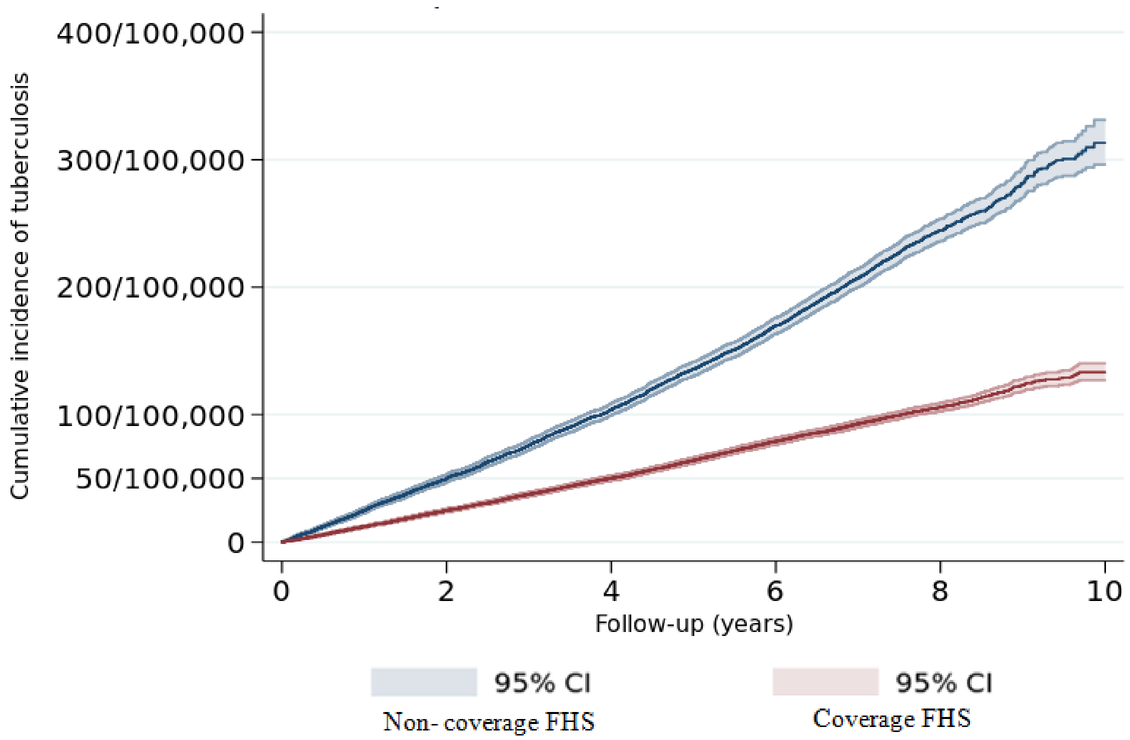

Web Figure 4. Cumulative Mortality of tuberculosis by percentage of FHS coverage (<10% and 100%) in the study cohort (Brazil, 2004 - 2013).

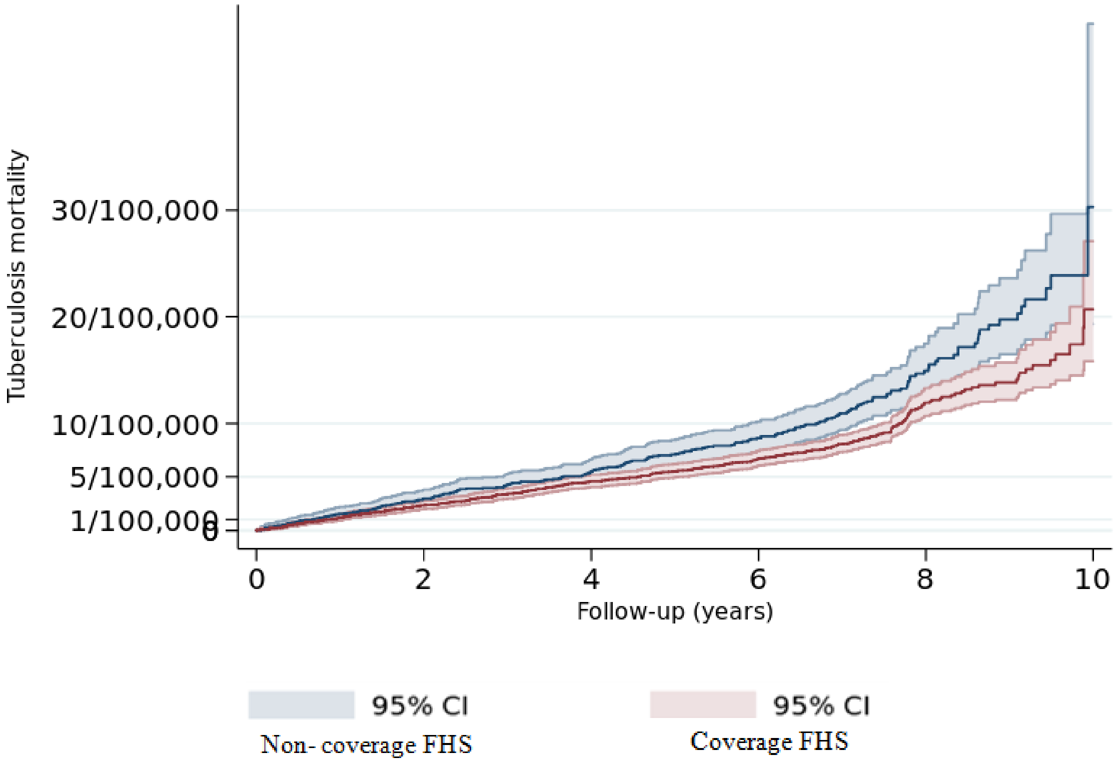

REFERENCES

1 Unified Registry | WWP. http://wwp.org.br/en/social-policy/unified-registry/ (accessed March 26, 2021).

2 Cidacs. https://cidacs.bahia.fiocruz.br/en/platform/cohort-of-100-million-brazilians (accessed March 26, 2021).

3 Barreto M, Alves A, Sena S, *et al.* Assessing the accuracy of probabilistic record linkage of social and health databases in the 100 million Brazilian cohort: IJPDS (2017) Issue 1, Vol 1:256 Proceedings of the IPDLN Conference (August 2016). *Int J Popul Data Sci* 2017; **1**. DOI:10.23889/ijpds.v1i1.276.

4 Ali MS, Ichihara MY, Lopes LC, *et al.* Administrative Data Linkage in Brazil: Potentials for Health Technology Assessment. *Front Pharmacol* 2019; **10**: 984.

5 Baptista EA, Abel GJ, Campos J. Internal migration in Brazil using circular visualization. *Reg Stud Reg Sci* 2018; **5**: 361–4.

6 Cain KC, Harlow SD, Little RJ, *et al.* Bias due to left truncation and left censoring in longitudinal studies of developmental and disease processes. *Am J Epidemiol* 2011; **173**: 1078–84.

7 Macinko J, Harris MJ. Brazil’s Family Health Strategy — Delivering Community-Based Primary Care in a Universal Health System. *N Engl J Med* 2015; **372**: 2177–81.
